# Cancer stem cells induced by chronic stimulation with prostaglandin E2 exhibited constitutively activated PI3K axis

**DOI:** 10.1038/s41598-022-19265-7

**Published:** 2022-09-17

**Authors:** Hideki Minematsu, Said M. Afify, Yuki Sugihara, Ghmkin Hassan, Maram H. Zahra, Akimasa Seno, Masaki Adachi, Masaharu Seno

**Affiliations:** 1 Laboratory of Nao-Biotechnology, Division of Medical Bioengineering, Graduate School of Natural Science and Technology, Okayama University, Okayama, 700-8530 Japan; 2 R&D Center, Katayama Chemicals Ind., Co. Ltd, Ina, Minoh, Osaka 562-0015 Japan; 3 Division of Biochemistry, Chemistry Department, Faculty of Science, Menoufia University, Shebin El Koum, 32511 Egypt; 4 Department of Biotechnology and Drug Discovery, Graduate School of Interdisciplinary Science and Engineering in Health Systems, Okayama University, Okayama, 700-8530 Japan

**Keywords:** Biochemistry, Cancer, Stem cells

## Abstract

Previously, our group has demonstrated establishment of Cancer Stem Cell (CSC) models from stem cells in the presence of conditioned medium of cancer cell lines. In this study, we tried to identify the factors responsible for the induction of CSCs. Since we found the lipid composition could be traced to arachidonic acid cascade in the CSC model, we assessed prostaglandin E2 (PGE2) as a candidate for the ability to induce CSCs from induced pluripotent stem cells (iPSCs). Mouse iPSCs acquired the characteristics of CSCs in the presence of 10 ng/mL of PGE2 after 4 weeks. Since constitutive Akt activation and pik3cg overexpression were found in the resultant CSCs, of which growth was found independent of PGE2, chronic stimulation of the receptors EP-2/4 by PGE2 was supposed to induce CSCs from iPSCs through epigenetic effect. The bioinformatics analysis of the next generation sequence data of the obtained CSCs proposed not only receptor tyrosine kinase activation by growth factors but also extracellular matrix and focal adhesion enhanced PI3K pathway. Collectively, chronic stimulation of stem cells with PGE2 was implied responsible for cancer initiation enhancing PI3K/Akt axis.

## Introduction

The number of patients and deaths of cancer is increasing year by year in the world, and it is the leading cause of death in Japan and the highest in the world. In recent years, the development of anti-cancer drugs including molecularly targeting drugs has progressed, and chemo- and radiation-therapy have become possible to temporarily treat many cancers. However, the population of the cells resistant to treatment will emerge as recurrence and/or metastasis so often that the number of cancer deaths should continue to increase^1^.

Cancer stem cells (CSCs) have been focused on recent years due to the contribution in cancer initiation^2^ and recurrence/metastasis^3^. Even if most of the tumor is removed by chemotherapy, recurrences are often found due to the drug resistance of CSCs. CSCs are the subpopulation within cancer tissues sharing similar characteristics of normal stem or progenitor cells such as self-renewal ability and multi-lineage differentiation to drive tumor growth and heterogeneity^4^. CSCs are hypothesized to be the top of the hierarchy of the cells and this idea is now being widely accepted^5^. Due to the heterogeneity, treatment of cancer seems to be difficult. Although the mechanism of CSC development has not been clear yet, elucidation of the mechanism will significantly contribute to the prevention, diagnosis, and treatment of cancer.

Based on the hypothesis of cancer-inducing niche^2^, our group developed CSC models from mouse induced pluripotent stem cells (miPSCs), which were cultured in the presence of conditioned media of Lewis lung carcinoma (LLC) cells^6^ or exosomes derived from the conditioned media for 4 weeks^7^. miPS-LLCcm cells are the model of CSCs prepared from miPSCs by the treatment with the conditioned medium of LLC cells. miPS-LLCcm cells were confirmed tumorigenic when subcutaneously transplanted and the primary cells from the tumor were designated as miPS-LLCcmP cells. Both miPS-LLCcm and miPS-LLCcmP cells were used as the control of converted cells in this study. However, the factors responsible for the conversion of miPSCs to CSCs has not yet been clarified. The identification of the responsible factors in the conditioned media of LLC cells may lead to the elucidation of the molecular mechanism of cancer initiation.

Although the relationship between chronic inflammation and cancer development has historically been suggested^2,8,9^, the relationship is not so much uncovered. This is partly because chronic inflammation varies and has been diagnosed personally leading to the difficulty to explain in general terms. On the other hand, we know to date various inflammation-related substances such as cell growth factors, cytokines including interleukins, and chemokines have been reported to be overexpressed in many cancer cell lines. Taking these into consideration, the presence of responsible factors for the induction of CSCs could be identified as inflammation-related substances in the conditioned medium of LLC cells. In this study, we found that the differences in lipid compositions between miPSCs and the induced CSCs. Based on this finding, we started analyses to explore the mechanism of CSC development attributed to the activation of PI3 kinase signaling pathway, which we previously identified to be enhanced in the induced CSCs^10,11^.

## Results

### Cancer related cells appeared rich in a source of inflammatory eicosanoids

**Figure 1:**
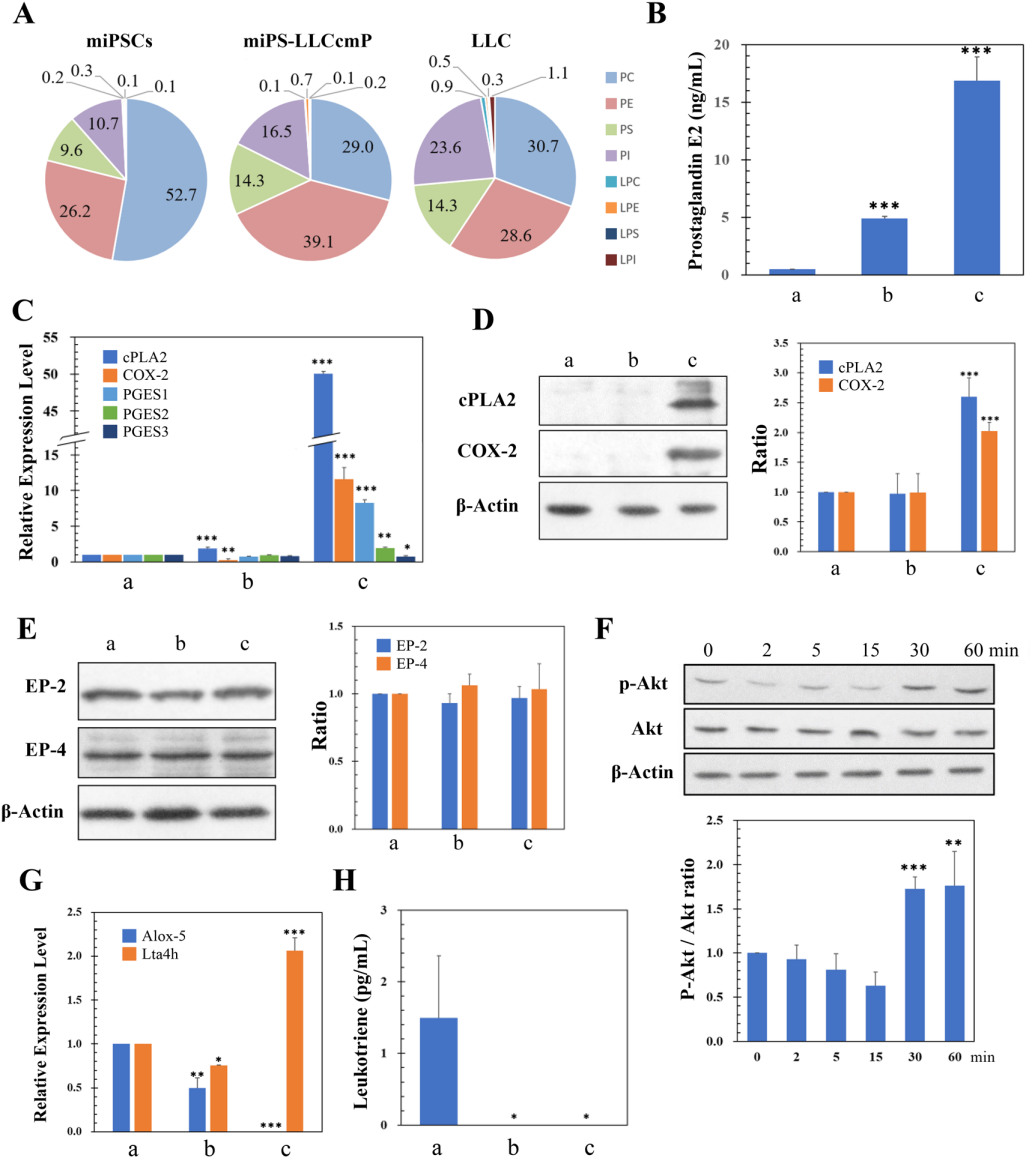
Comparison of the characters between miPSCs, miPS-LLCcmP cells and LLC cells. (**A**) Comparison of the glycerophospholipid composition ratio. (**B**) Comparison of PGE2 production by ELISA. a: miPSCs, b: miPS-LLCcmP, c: LLC. Data are obtained from three independent experiments and plotted as means ± SD.***p < 0.01. (**C**) Comparison of relative expression of gene related with PGE2 synthesis. a: miPSCs, b: miPS-LLCcmP, c: LLC. Data are obtained from three independent experiments and plotted as means ± SD. *p < 0.1, **p < 0.05, ***p < 0.01. (**D**) Comparison of the expression of cPLA2 and COX-2 by Western blotting. A representative blot is shown at left. Densitometric analysis of the blots from three independent experiments by ImageJ is shown at right. a: miPSCs, b: miPS-LLCcmP, c: LLC. Data are plotted as means ± SD. ***p < 0.01. (**E**) Comparison of the expression of EP-2 and EP-4 by Western blotting. A representative blot is shown at left. Densitometric analysis of the blots from three independent experiments by ImageJ is shown at right. a: miPSCs, b: miPS-LLCcmP, c: LLC. Data are plotted as means ± SD. (**F**) Time course change of the phosphorylation of Akt in miPSCs. A representative blot is shown at left. Densitometric analysis of the blots from three independent experiments by ImageJ is shown at right. Data are plotted as means ± SD. **p < 0.05, ***p < 0.01. (**G**) Comparison of the expression of Alox-5 and Lta4h by RT-qPCR. a: miPSCs, b: miPS-LLCcmP, c: LLC. Data are obtained from three independent experiments and plotted as means ± SD. *p < 0.1, **p < 0.05, ***p < 0.01. (**H**) Comparison of the production of LTB4 by ELISA. a: miPSCs, b: miPS-LLCcmP, c: LLC. Data are obtained from three independent experiments and plotted as means ± SD. *p < 0.1. See details in “Materials and methods” section.

LLC and miPS-LLCcmP cells showed the composition of phosphatidylserine (PS) 14.3% and phosphatidylinositol (PI) 23.6 and 16.5%, respectively, whereas PS was 9.6 and PI 10.7% in miPSCs. And the ratio of phosphatidylcholine (PC) is in LLC and miPS-LLCcmP cells were 30.7 and 29.0% whereas it was 52.7% in miPSCs (Fig. 1A). Thus, PS and PI were relatively higher, and PC was lower in the cancer related cells when compared with normal stem cells. The composition of glycerophospholipids such as PI, PS and PC in cancer cells is reported to significantly be different from that in normal cells^12^. The increased ratio of PI could be traced to the conversion of miPSCs into CSCs because the PI-metabolic system is involved in the development of cancer^13^. Furthermore, most of the fatty acids at the 2-position of PI are arachidonic acid^14,15^. Generally, arachidonic acid is used in vivo as a part of prostaglandins and leukotrienes, so-called eicosanoids, which are closely related to inflammation and cancer^16^. We hypothesized the composition ratio of PI in miPS-LLCcmP cells and LLC cells higher than in miPSCs should imply the involvement of inflammatory eicosanoids in the conversion of miPSCs into miPS-LLCcmP cells.

### Presence of prostaglandin E2 in the conditioned medium of LLC cells

Since the major candidate of prostaglandin involved in inflammation was prostaglandin E2 (PGE2), the amount of PGE2 in the conditioned medium (CM) of LLC cells, which was supposed effective in the conversion of miPSCs into CSCs, was quantified by ELISA. As the result, LLC cells produced 16.9 ng/mL of PGE2, which was approximately 30 times higher than the amount of 0.5 ng/mL produced by miPSCs (Fig. 1B). miPS-LLCcmP cells produced 4.9 ng/mL of PGE2 implying that the converted cells acquired the ability to produce PGE2.

Further, we assessed the expression of cytoplasmic phospholipase A2 (cPLA2), which digests PI to excise arachidonic acid, cyclooxygenase (COX-2), which is induced in inflammation and catalyses cyclization of C8–C12 of arachidonic acid, and prostaglandin E synthase (PGES) 1 by RT-qPCR (Fig. 1C). As the results, LLC cells overexpressed 50, 11 and 8 folds of cPLA2, COX-2 and PGES1, respectively, more than miPSCs while those in miPS-LLCcmP cells were almost equivalent to those in miPSCs. The results of Western Blot for cPLA2 and COX-2 were consistent with those of rt-qPCR (Fig. 1D).

The expression of PGE2 receptors was then assessed in miPSCs in order to confirm that PGE2 could stimulates miPSCs to activate the cytoplasmic signalling. EP-2 and EP-4, which were closely related to cancer among PGE2 receptors, were found expressed in miPSCs as well as miPS-LLCcmP cells and LLC cells (Fig. 1E).

Since PI3K/Akt pathway was reported to be activated through EP-2 or EP-4, both of which were subtypes of G-protein coupled receptor (GPCR) stimulated by PGE2^17–19^, the phosphorylation of Akt was assessed in miPSCs by the stimulation of PGE2 (Fig. 1F). As the result, Akt was phosphorylated by 10 ng/mL of PGE2 in miPSCs showing the time course dependent manner with the peak at 60 min from the stimulation.

Leukotriene B4 (LTB4) is a biproduct of arachidonic acid and also known as one of the inflammatory eicosanoids like PGE2. The enzymes responsible for the synthesis are arachidonate 5-lipoxigenae (Alox-5) and leukotriene A4 hydrolase (LTA4h). Since LTB4 is considered closely related with cancer^16^, the expression of these two enzymes in the three cells was assessed by RT-qPCR. However, the expression level of Alox-5 was not detectable in LLC cells while LTA4h was detected indicating the synthesis of LTB4 should be difficult (Fig. 1G). Further, the quantity of LTB4 in the CM of LLC cells was not detectable by ELISA, of which sensitivity was lower than 1 pg/mL (Fig. 1H). Taking all these data into consideration, PGE2 was supposed to be a factor responsible for the conversion of miPSCs into CSCs.

### Evaluation of PGE2 to convert miPSCs to CSCs

**Figure 2:**
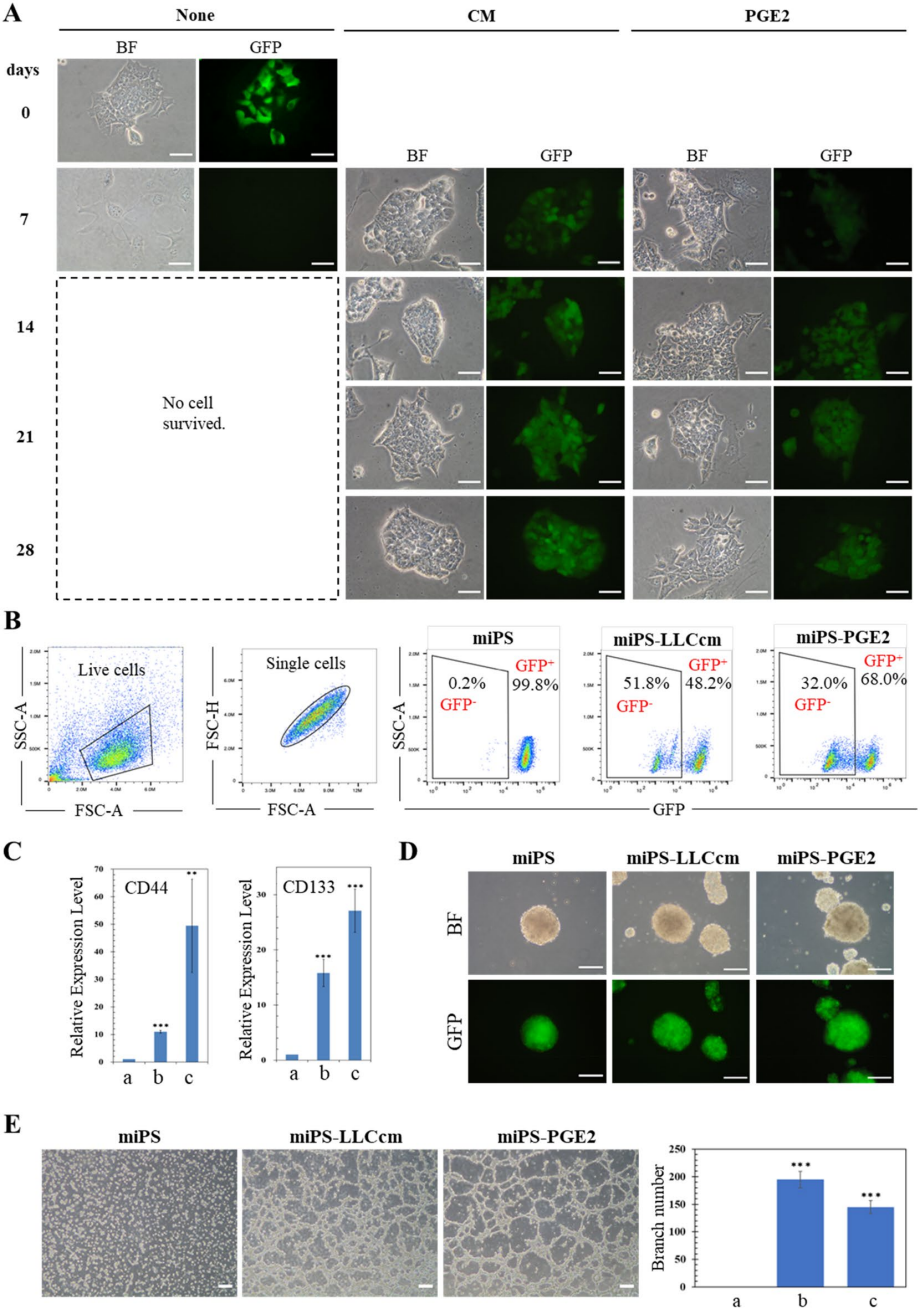
Evaluation of the effect of PGE2 on miPSCs. (**A**) Time course change of morphology during 4 weeks of treatment. The images of the cells before treatment are put at the left as the control of the start point with GFP positive cells. BF, bright field; GFP, green fluorescence. Scale bar 50 µm. (**B**) Flow cytometric analysis for the GFP^+^ population in miPSCs, miPS-LLCcm cells and miPS-PGE2 cells. (**C**) Comparison of the expression of CD44 and CD133 between miPSCs, miPS-LLCcm cells and miPS-PGE2 cells by RT-qPCR. a: miPSCs, b: miPS-LLCcm, c: miPS-PGE2. Data are plotted as means ± SD. *p < 0.1, **p < 0.05, ***p < 0.01. (**D**) Comparison of the sphere formation of miPSCs, miPS-LLCcm cells and miPS-PGE2 cells. Scale bar 200 µm. (**E**) Tube formation assay on miPSCs, miPS-LLCcm cells and miPS-PGE2 cells (left). Scale bar 200 µm. Branching point analysis by ImageJ (right). a: miPS, b: miPS-LLCcm, c: miPS-PGE2. Data are plotted as means ± SD. ***p < 0.01.

Since the concentration of PGE2 was almost 20 ng/mL in the CM of LLC cells and the CM was diluted two-fold when miPSCs were previously converted into miPS-LLCcm cells, miPSCs were cultured in the presence of 10 ng/mL of PGE2 for 4 weeks without leukaemia inhibitory factor (LIF) which was required to maintain the stemness of miPSCs in the standard culture condition as described in the Materials and Methods section. As the result, miPSCs survived as those cultured in the presence of CM of LLC cells while they died within 1 week after culturing without LIF or PGE2 (Fig. 2A). These cells were designated as miPS-PGE2 cells, which kept GFP expressed under the control of Nanog promoter indicating undifferentiated condition of the cells in the same manner as demonstrated in miPS-LLCcm cells^6^ (Fig. 2A). Flow cytometric analysis showed that GFP^+^ population in miPS-PGE2 cells was 68% while that in miPS-LLCcm cells was 48% indicating the resultant cells of the conversion were the mixture of differentiated and undifferentiated cells when they were cultured in adhesive condition (Fig. 2B). It was worthwhile noticing that miPS-PGE2 cells acquired the expression of CSC markers such as CD44 and CD133 (Fig. 2C).

Since the sphere-forming assay was first introduced as a functional approach for studying adult stem cells^20^ and has been widely used to evaluate the stem properties of proposed CSC populations^21–23^ miPS-PGE2 cells were assessed for the self-renewal potential by the sphere formation in a dish with low attachment surface. As the result, miPS-PGE2 cells formed spheres under non-serum condition indicating self-renewal potential in the same manner as demonstrated in miPS-LLCcm cells (Fig. 2D). The potential of differentiation into vascular endothelial cells (ECs) in miPS-PGE2 cells was assessed for the tube formation on Matrigel. The tube-forming capacity of the cells was quantified by the number of branching points (Fig. 2E). As the result, miPS-PGE2 exhibited the potential of differentiation as well as that of miPS-LLCcm cells.

**Figure 3:**
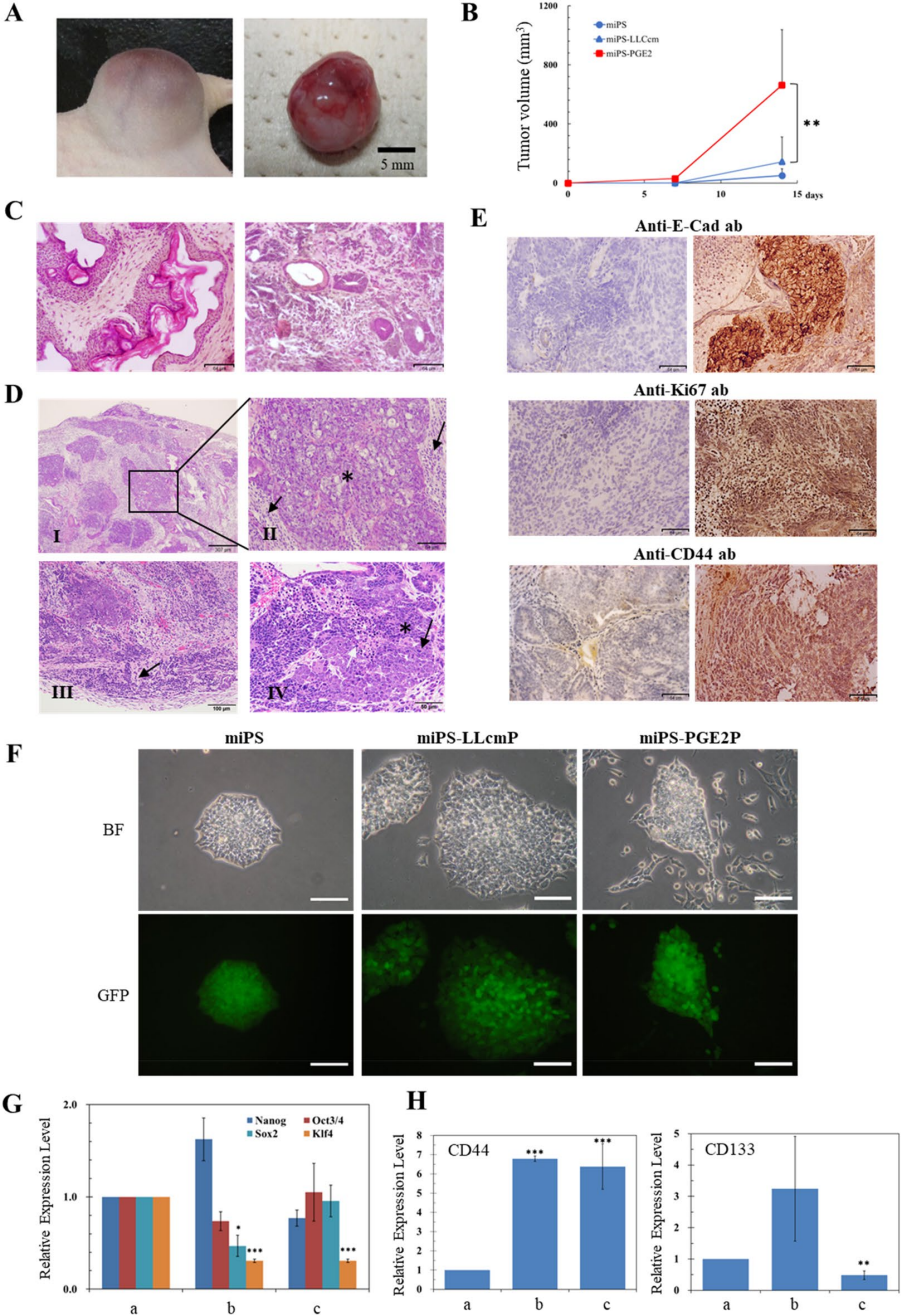
Analyses of tumorigenic potential in miPS-PGE2 cells. (**A**) Tumor derived from miPS-PGE2 cells in s.c. (left) and excised (right). Scale bar 5 mm. (**B**) Time course change of the tumor size. Data are plotted by means + SD. **p < 0.05 (**C**) Representative images from two different field of miPSCs derived teratoma after four weeks of injection as a control showing a phenotype with various normal germ layers, including squamous epithelium, skeletal muscle, cartilage, and benign glandular epithelium. (**D**) Representative images of miPS-PGE2 derived malignant tumor after four weeks of injection (**C**,**D**) H&E staining. I, low magnification. II, maginification of the part squared in I. III and IV, Different fields of the section. Asterisks in II and IV depict severe nuclear atypia. Black arrows in II and IV depict mild nuclear atypia. Black arrow in III depicts invasion. White arrow in IV depicts necrosis. Scale bars 307 (top left), 64 (top right), 100 (bottom left), 50 (bottom right) µm. (**E**) Immunohistochemistry of the tumor in A. The tumor was stained with antibodies against E-cadherin, Ki67 and CD44. Sections from miPSC derived teratoma (left column) and those from miPS-PGE2 derived malignant tumor (right column) Scale bars 64 µm. (**F**) Adhesive culture of miPSCs and the primary cells derived from miPS-LLCcm and miPS-PGE2 cells. BF, bright field. GFP, fluorescence from GFP. Scale bar 100 µm. (**G**) Comparison of the expression of Stemness marker between miPSCs (a), miPS-LLCcmP cells (b) and miPS-PGE2P cells (c) by RT-qPCR. Data were plotted as means ± SD. *p < 0.1, **p < 0.05, ***p < 0.01. (**H**) Comparison of the expression of CD44 and CD133 between miPSCs (a), miPS-LLCcmP cells (b) and miPS-PGE2P cells (c) by RT-qPCR. Data are plotted as means ± SD. **p < 0.05, ***p < 0.01.

Subcutaneously transplanted miPS-PGE2 cells formed tumors in nude mice (Fig. 3A). It is worthwhile noticing that the converted cells exhibited significantly rapid tumor growth when compared to miPSCs, which developed benign teratoma, and miPS-LLCcm cells, which developed malignant tumor. The volume of tumors from miPS-PGE2 cells reached to an average of 600 mm^3^ in less than 2 weeks while those from miPS-LLCcm cells required at least 3 weeks to reach to the same size (Fig. 3B). While histological features of teratoma derived from miPSCs showed no malignancy (Fig. 3C), the xenografts derived from miPS-PGE2 cells showed high mitotic figures and a high degree of atypia (Fig. 3D I, II), which were similar to those of the malignant tumors developed from miPS-LLCcm cells^6^. The secondary tumor by the injection of miPS-PGE2P cells showed infiltration into the surrounding muscular layer and high mitotic figures and nuclear atypia (Fig. 3D III, IV). Simultaneously, immunohistochemistry of the tumor derived from miPS-PGE2P cells showed strong immunoreactivity to anti-E-cadherin, -Ki67, and -CD44 antibodies indicating high proliferation rate with CSC subpopulation as well as heterogeneous intercellular adherence while teratoma section did not show any of these immunoreactivities (Fig. 3E).

### Primary culture of miPS-PGE2 derived tumor showed characteristics of CSCs

**Figure 4:**
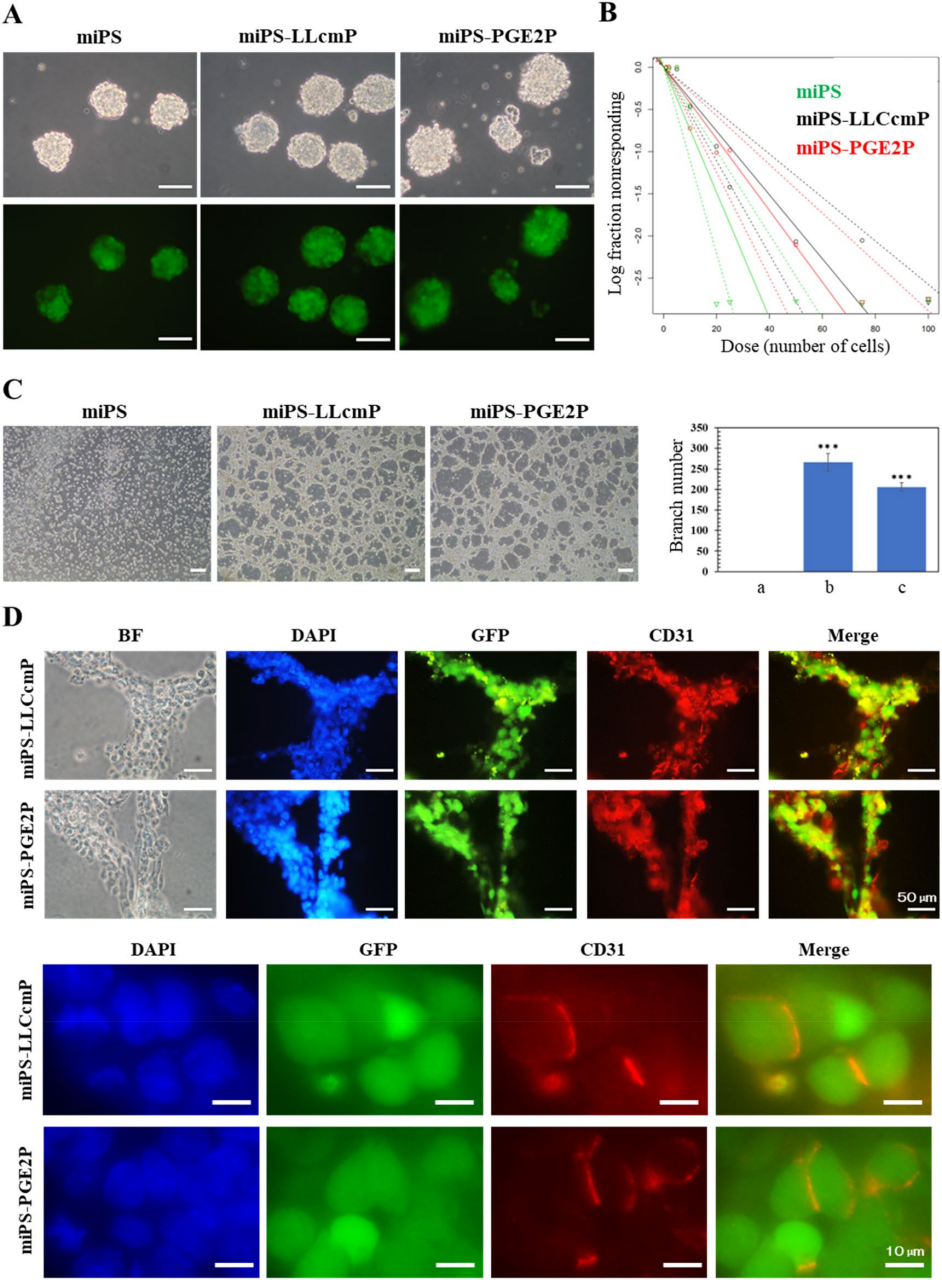
Self-renewal and differentiation potential of miPS-PGE2P cells compared with miPS-LLCcmP cells. (**A**) Typical spheres of the cells in non-adherent condition. Scale bar 100 µm. (**B**) ELDA for sphere formation miPS-PGE2P cells (Green), miPS-LLCcmP cells (red) and miPSCs (black). (**C**) Tube formation assay. Branching point analysed by ImageJ. a: miPS, b: miPS-LLCcmP, c: miPS-PGE2P. Data are plotted as means ± SD. ***p < 0.01 (**D**) Immunofluorescent analyses of tubes in (**C**). *BF* bright field, *DAPI* staining for nucleus, *GFP* fluorescence of GFP, *CD31* staining with anti-CD31 antibody labelled with Alexa fluor 555. Scale bars 50 (top) and 10 µm (bottom).

Primary culture of a tumor developed from miPS-PGE2 cells was designated as miPS-PGE2P. miPS-PGE2P cells colonized and proliferated on an adhesive culture dish in the same manner as miPS-LLCcm cells (Fig. 3F). miPS-PGE2P cells sustained the expression of GFP, which could be traced back to the injected miPS-PGE2 cells and showed two different subpopulations of GFP^+^ and GFP^-^ cells representing the undifferentiated and differentiated subpopulation, respectively. GFP^-^ cells exhibited fibroblast-like morphology apparently supporting the microenvironment of the GFP^+^ cells that are CSCs. Regarding to the stemness, miPS-PGE2P cells sustained the expression of stem cell markers such as Nanog, Oct3/4 and SOX2 (Fig. 3G) while the level of KLF4 was significantly downregulated as it was in miPS-LLCcm cells. As for the markers of CSC, miPS-PGE2P cells showed the expression of CD44 and CD133 whereas the expression of CD44 was as the same level as that in miPS-LLCcm cells but higher than that in miPSCs (Fig. 3H). miPS-PGE2P cells formed spheres in the serum free condition (Fig. 4A). The extreme limiting dilution assay showed that the self-renewal potential of miPS-PGE2 cells was significantly higher than that of miPS-LLCcmP cells (Fig. 4B). The potential of differentiation into ECs in miPS-PGE2P cells was assessed on Matrigel and evaluated by the tube formation (Fig. 4C). miPS-PGE2P cells were found sustaining the differentiation potential as shown in miPS-PGE2 and miPS-LLCcmP cells. The tube structures were most extensive during 12–24 h after seeding. There was no significant difference in the number of tubes and branch lengths between miPS-PGE2 and miPS-LLCcm cells after 24 h. The differentiation was simultaneously assessed by immunofluorescence staining for CD31, which serves as phenotypic and functional markers of ECs (Fig. 4D). miPS-PGE2 cells expressed CD31 in the tubes together with GFP exhibiting different subpopulations of “CD31^+^/GFP^-^ (red)”, and “CD31^−^/GFP^+^ (green)” and “CD31^+^/GFP^+^ (yellow)”. This result indicated that the tumor angiogenesis might occur by CSCs sequentially differentiating from undifferentiated phenotype (green) to differentiated phenotype (red) through mid-stage (yellow) and that the tube structures were finally composed of heterogeneous cells, which would partly explain the heterogeneity in a tumor tissue.

### PI3K/Akt signalling in miPS-PGE2 and miPS-PGE2P cells

**Figure 5:**
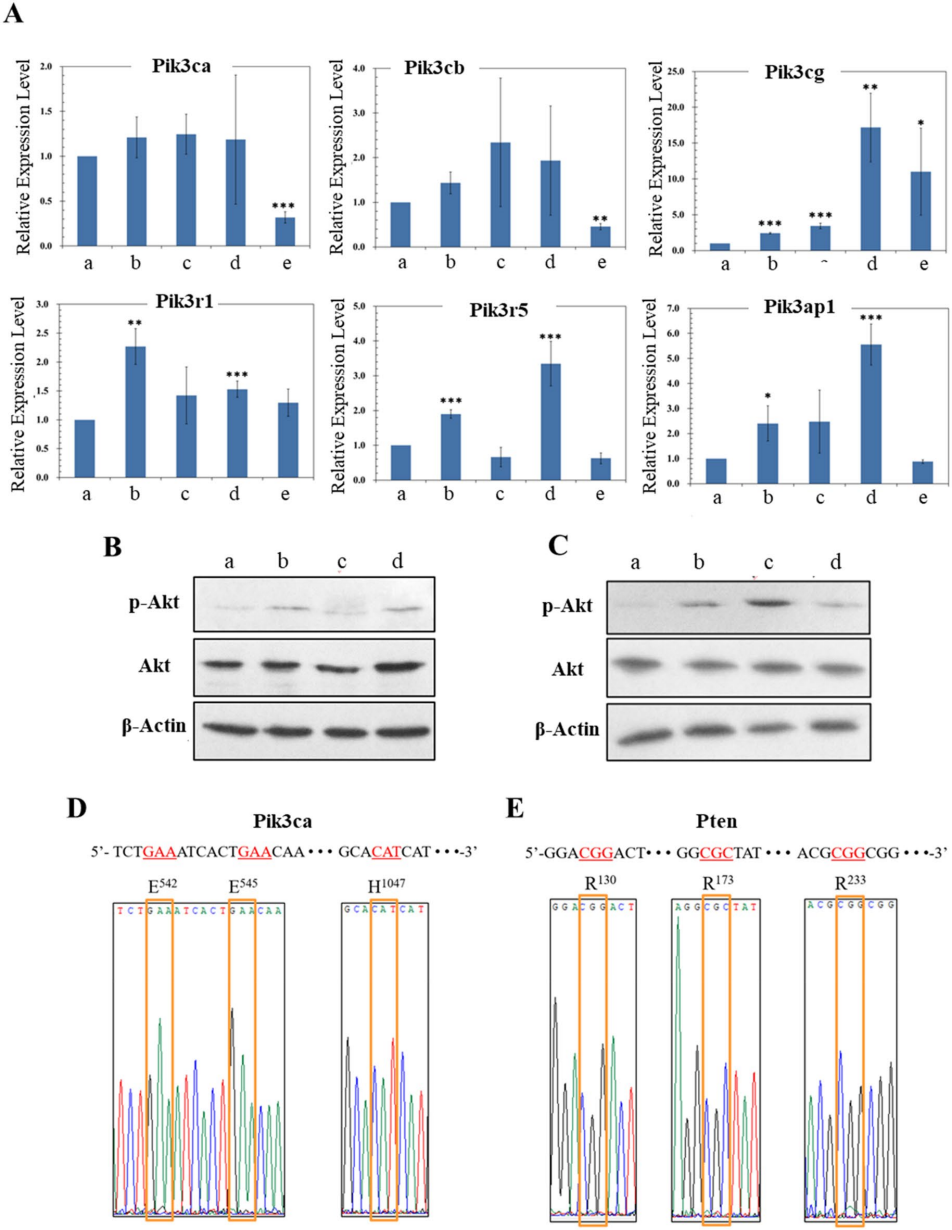
Analyses of PI3K/Akt signalling potential of miPS-PGE2P cells. (**A**) RT-qPCR analyses of pik3 related genes expression. a: miPSCs, b: miPS-LLCcm, c: miPS-PGE2, d: miPS-LLCcmP, e: miPS-PGE2P. Data are plotted as means ± SD. *p < 0.1, **p < 0.05, ***p < 0.01. (**B**,**C**) Western blotting analysis of the phosphorylation of Akt. (**B**) Cells after conversion of iPSCs. a: miPSCs -LIF, b: miPSCs + LIF, c: miPS-LLCcm, d: miPS-PGE2. (**C**) Primary cells derived from tumors of miPS-LLCcm and miPS-PGE2 cells. a: miPSCs -LIF, b: miPSCs + LIF, c: miPS-LLCcmP, d: miPS-PGE2P. (**D**,**E**) DNA sequence spots of oncogenic mutations in pik3ca and PTEN genes were analysed in miPS-PGE2P cells. The sequencing charts corresponding to each part of sequences are shown together with normal sequence and translated amino acids on the top. (**D**) pi3kca gene (**E**) PTEN gene. The ID numbers of the template sequences are BC089038.1 in EMBL and NM_008960.2 in GenBank. Each codon shown in red is the part subjected to occasional oncogenic mutation in cancer (COSMIC < https://cancer.sanger.ac.uk/cosmic >).

Previously, our group reported that PI3K/Akt signaling was activated in miPS-LLCcm cells^10^. Following this information, we assessed the activation of PI3K/Akt signaling in miPS-PGE2 cells. As the results of RT-qPCR, the expression of Pik3cg was significantly upregulated in miPS-PGE2 cells when compared to that in miPSCs as found in miPS-LLCcm cells (Fig. 5A). Regarding to the activation of PI3K/Akt signaling, the phosphorylation of Akt was assessed by Western Blotting. As a result, the constitutive phosphorylation of Akt in miPS-PGE2 cells was recognized as found in miPS-LLCcm cells and higher than that in miPSCs without LIF (Fig. 5B,C). Among PI3K, oncogenic frequent mutations in the Pik3ca gene have been reported in human cancer^13^. The typical amino acid changes due to the mutations are E542K, E545K, and H1047R. However, no mutations were found in the Pik3ca gene in miPS-PGE2 cells by the cDNA sequence analysis (Fig. 5D). It is conceivable that upregulation of PI3K level enhances the product of phosphatase of phosphatidyl inositol 3,4,5 phosphate (PIP3) triggerring PI3K/Akt pathway toward carcinogenesis^24,25^. Since PI3K/Akt pathway is negatively regulated by phosphatase and tensin homologue (PTEN), which is a lipid hydrolytic enzyme specific to PIP3, PTEN is considered as the tumor suppressor gene. Three major mutations PTEN gene resulting in the amino acid changes of R130L, R173C and R233X, where X means anonymous, have been reported to inactivate PTEN in human cancers^26–28^. Accordingly, we assessed the expression of PTEN and cDNA sequence (Supplementary Fig. S1 and Fig. 5E). The expression of PTEN was lower in miPS-PGE2 cells than in miPSCs. And there were no mutations in the three mainly reported sites in the PTEN gene in miPS-PGE2 cells.

**Figure 6:**
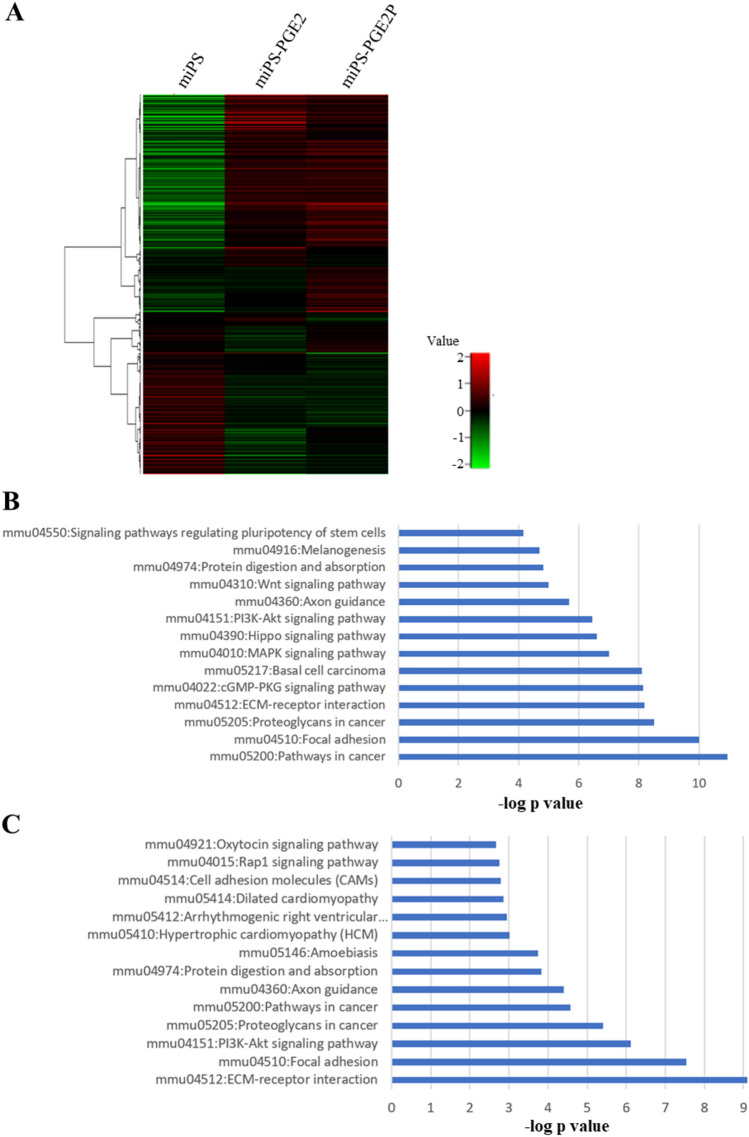
Transcriptome analysis of miPS-PGE2 and miPS-PGE2P cells. (**A**) A heatmap of gene expression profiles between miPSCs, miPS-PGE2 and miPS-PGE2P cells. (**B**,**C**) The results of KEGG pathway analysis showing the top enriched pathways compared to miPSCs. (**B**) miPS-PGE2 cells. (**C**) miPS-PGE2P cells.

The RNA from converted miPS-PGE2 cells, primary cultured miPS-PGE2P cells and miPSCs were subjected to the RNA-seq and the gene expression profiles were compared as a heat map using iDEP (Fig. 6A). The upregulated genes depicted in red when compared to miPSCs in the converted cells and the primary cells were respectively picked up and applied to Kyoto Encyclopedia of Gene and Genomes (KEGG) pathway analysis. The analysis revealed that top enriched pathways in the converted cells and the primary cells were including those related to cancer, stemness, and cell-to-cell or cell-to-matrix interactions (Fig. 6B,C). The Parametric Gene Set Enrichment Analysis (PGSEA) showed commonly activated KEGG pathways between converted and primary cells when compared with miPSCs were “mmu05200: Pathways in cancer”, “mmu04510: Focal adhesion”, “mmu04390: Hippo signaling pathway”, “mmu04512:ECM-receptor interaction”, “mmu04310:Wnt signaling pathway”, and “mmu04151:PI3K-Akt signaling pathway”, which were apparently related with cancer. The genes categorized in the pathways were picked up and checked their normalized counts. The genes of which counts were upregulated in both miPS-PGE2 and miPS-PGE2P cells were further picked up and summarized in the supplementary Table S1. From the Table S1, the genes with extensive upregulation were collagen type I α1 (COL1A1), collagen type IV α1 (COL4A1), collagen type IV α2 (COL4A2), filamin B (FLNB), GLI family zinc finger 2 (GLI2), insulin-like growth factor 1 receptor (IGF1R), integrin subunit αV (ITGAV), vinculin (VCL). VCL is considered as adaptor supporting integrin to connect with actin filament^29^. FLNB is known as an actin-binding protein^30^. On the other hand, collagens appear to enhance the extracellular matrix (ECM). Especially, COL1A1 may play supportive function to cancer associated fibroblasts in vivo^31^. Collectively, the cancer stem cells appear to be strengthened by the cytoskeleton together with the ECM connected through integrin molecule.

### miPS-PGE2P cells maintain CSC characteristics without exogenous PGE2

**Figure 7:**
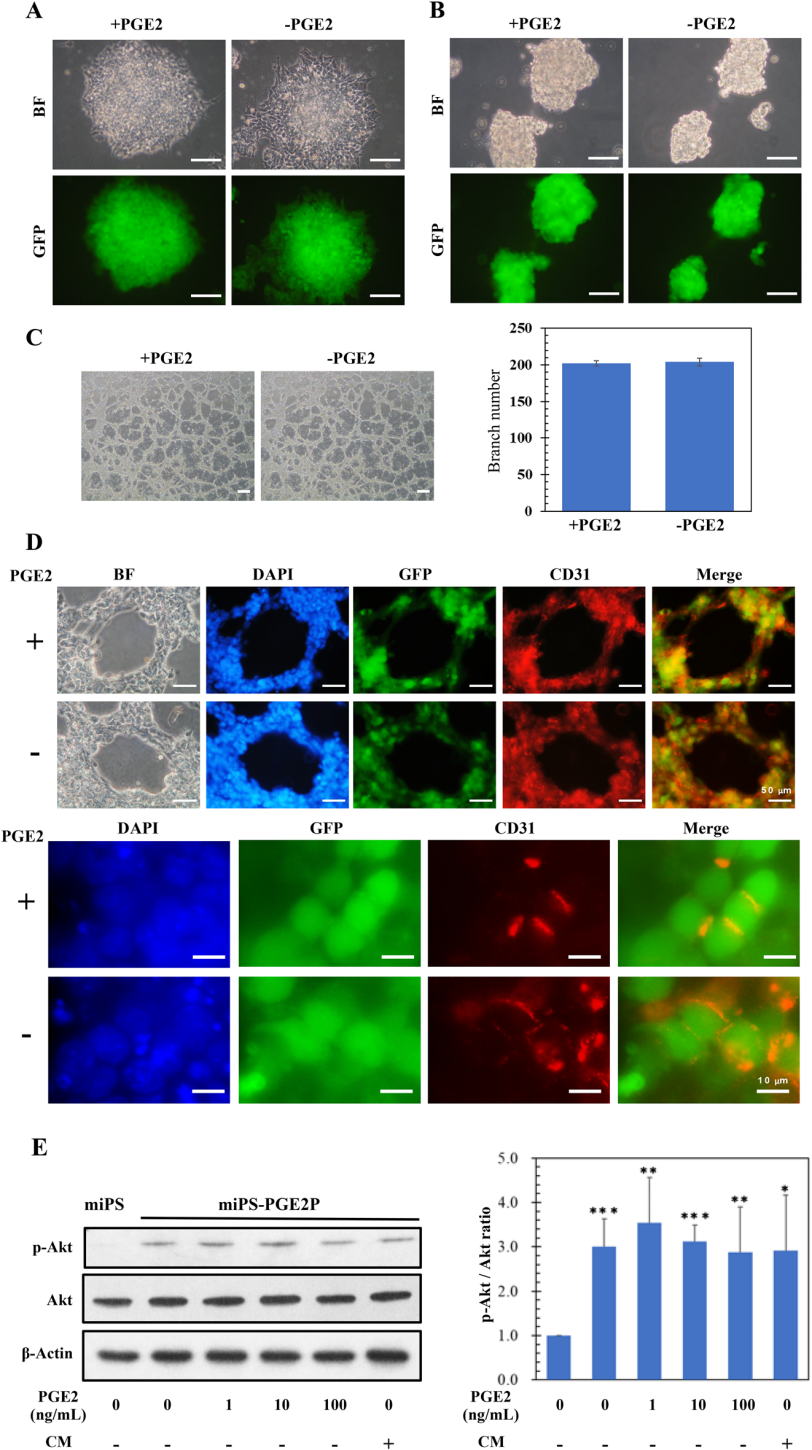
Characterization of miPS-PGE2P cells in the presence or absence of PGE2. (**A**) Adhesive culture. Scale bar 100 µm. (**B**) Non-adhesive culture. Scale bar 100 µm. (**C**) Tube formation assay (left). Scale bar 100 µm. Branching point analysis by ImageJ. (right) Data are plotted as means ± SD. There was no significance between the data. (**D**) Immunofluorescent analyses of tubes in C. BF, bright field. DAPI, staining for nucleus. GFP, fluorescence of GFP. CD31, staining with anti-CD31 antibody labelled with Alexa fluor 555. Scale bars 50 µm (top) and 10 µm (bottom). (**E**) Western blotting analysis of the phosphorylation of Akt. A representative blot is shown at left. Densitometric analysis of the blots from three independent experiments by ImageJ is shown at right. Data are plotted as means ± SD. *p < 0.1, **p < 0.05, ***p < 0.01.

The dependency of CSC characters on PGE2 in the primary culture derived from tumor of miPS-PGE2 cells, miPS-PGE2P cells, was assessed. First of all, miPS-PGE2P cells were divided into two groups of culture with/without PGE2 and cultured for 2 weeks. As the result, miPS-PGE2P cells exhibited the growth in adhesive condition, sphere formation and differentiation into vascular endothelial cells in the manner independent of PGE2 (Fig. 7A–D) (Supplementary Fig. S7). Simultaneously, the phosphorylation of Akt was assessed in the presence or absence of PGE2 in miPS-PGE2P cells. And Akt was found phosphorylated even without PGE2 (Fig. 7E). Collectively, miPS-PGE2P cells could be concluded to acquire the CSC signatures independent of exogenous PGE2. Although the Akt phosphorylation in miPS-PGE2P cells was independent of exogenous PGE2 stimulation, the autocrine dependency of the cells on PGE2 is still not ruled out. Therefore, the production of PGE2 in miPS-PGE2 and miPS-PGE2P cells was assessed by ELISA. The amount of PGE2 produced in the conditioned medium of both cells was estimated to be approximately 0.3 ng/mL which is almost at the same level as that in the conditioned medium of miPSCs (Supplementary Fig. S2). Since the level of PGE2 in the conditioned medium of LLC cells was approximately 17 ng/mL (Fig. 1B), the level of PGE2 produced by miPS-PGE2P cells is almost 50 to 60 folds less than that by LLC cells. In this context, miPS-PGE2 cells could be independent of the exogenous PGE2 to maintain the CSC signatures.

## Discussion

Cancer initiation is an important issue from the viewpoint of cancer prevention and therapy, but it is still controversial. Chronic inflammation is sometimes tied to the development of cancer. Chronic inflammation could be traced to a lot of causes which have been suspected to raise the risk for certain cancers. Some infections can trigger inflammation affecting surrounding cells. If the inflammation continues for long period, it is considered to eventually lead to cancer. Autoimmune disorders such as collagen disease, ulcerative colitis, type II diabetes and so on will induce chronic inflammation. Obesity is forming fat tissue with angiogenesis expressing inflammatory factors such as tumor necrosis factor alpha (TNF-alpha), monocyte chemoattractant protein-1 (MCP-1), interleukin 6 (IL-6) and so on which enhance chronic inflammation and will lead to higher risks of cancer. Other factors such as poor sleep quality and heavy stress may also be elucidated to contribute to chronic inflammation. Considering these events, chronic inflammation always involves continued immune responses attacking invaders such as with interleukins, cytokines, chemokines, and reactive oxygen species (ROS) impairing tissues followed by tissue repairing with growth factors and metabolisms of sugars and fatty acids. The cells, which are exposed to these microenvironments so long period will get used to respond to change phenotypes initiating cancer, are so plastic enough that they should be a kind of stem like cells or progenitor cells with differentiation potential.

We are proposing the factors related with inflammation that induce CSCs from stem like cells might be an important clue in elucidating the mechanism of cancer initiation. In this study, we focused on lipid metabolismand found the ratio of PS and PI in LLC cells and miPS-LLCcm cells were higher than that in miPSCs (Fig. 1). This information was a clue to link the lipid metabolism with arachidonic acid cascade, which is closely related with inflammation. Then we demonstrated PGE2 could convert miPSCs into CSCs. The resultant miPS-PGE2 cells exhibited the CSC characters of self-renewal maintaining stemness, differentiation and malignant tumorigenesis (Figs. 4 and 5). Simultaneously, Akt was constitutively activated in miPS-PGE2 cells (Fig. 7). It is conceivable that the continuous stimulation of EP-2/4 with PGE2 on miPSCs lead to the constitutive activation of Akt in the conversion of miPSCs to CSCs. We previously reported that Akt was constitutively activated in miPS-LLCcm cells with epigenetically overexpressed pik3r5 and pik3cg genes^10^. In this study, we also found the over expression of pik3cg in miPS-PGE2 cells. However, the typical oncogenic mutations of the Pik3ca gene in miPS-PGE2 cells was not found (Fig. 5D). In this context, pik3cg could be responsible for the constitutive activation of PI3K/Akt signalling in miPS-PGE2 cells.

On the other hand, the expression level of PTEN was lower in miPS-PGE2 cells than in miPSCs (Supplementary Fig. S1) and no typical mutation was found in the nucleotide sequence of PTEN in miPS-PGE2 cells (Fig. 5E). Therefore, the constitutive activation of PI3K/Akt signalling pathway in miPS-PGE2 cells could possibly be elucidated by the autocrine stimulation of EP-2/4 by PGE produced by miPS-PGE2 cells. However, PGE2 was not detected in the conditioned medium of miPS-PGE2 cells and miPS-PGE2 cells maintained the properties of cancer stem cells in a PGE2-independent manner. And there was no autocrine loop of PGE2 found in miPS-pGE2 cells (Fig. 7, Supplementary Fig. S2). Collectively, constitutive activation of PI3K/Akt signalling pathway in miPS-PGE2 cells could be due to the hypomethylation of genes resulting in the overexpression of pik3cg coupled with the attenuated expression PTEN.

As the conclusion, chronic exposure of PGE2 to miPSCs will continuously stimulate EP-2/4 receptors activating PI3K resulting in the epigenetic upregulation of pik3 genes by hypomethylation of CpG islands leading the constitutive activation of PI3K/Akt signalling pathway in miPS-PGE2 cells. These results suggest that PGE2 should be considered as one of the candidate factors related with inflammation which could initiate cancer converting stem cells or progenitor cells. Although the detailed molecular mechanism remains unclear, the concept that PGE2 signal pathway could promote carcinogenesis has been described^32^. It has also been reported that PGE2 promotes colorectal cancer stem cell expansion and metastasis in mice^33^. Overexpression of COX-2 has been well known to induce colorectal cancer and, on the other hand, aspirin as a selective inhibitor of COX-2 has been expected to be effective for cancer prevention^34^. Actually, in the clinical cases aspirin doses have experienced significant reduce of cancer cases whereas the side effects should be taken care of^35,36^. In this context, the precise mechanism of conversion of stem cells into CSCs by PGE2 should be investigated further. The pathway analysis performed in this study showed that not only one but several pathways are found enriched in the cells exposed to PGE2. Moreover, it suggests that the crosstalk between these pathways is creating new cell phenotype where iPSCs maintain stemness and acquire malignancy. Although, the enriched pathways are well known for its roles in carcinogenesis and stemness maintenance, our data here shows a unique network of pathways upregulated in CSCs converted from normal cells. PI3K/Akt pathway appears to form cytoskeleton interacting with the extracellular matrices^37^. It may be worthwhile noticing that GLI2, which is known as the mediator of Sonic hedgehog (Shh) signaling supporting the maintenance of CSCs^38^, and IGFR1, which is a tyrosine kinase activating the cytoplasmic signals closely related with the growth stimulation^39^, are found significantly upregulated from the bioinformatic analyses.

We previously reported that chronic exposure to FGF2 converts iPSCs into cancer stem cells with an enhanced integrin/focal adhesion/PI3K/AKT axis^37^. PGE2 may have a role of induction of inflammation while FGF2 may have the role of wound healing after inflammation. The functional roles of these two factors could be a complement each other enhancing the feed-back and -forward in chronic inflammation repeating tissue repair. Taking these into consideration, the CSC model established from miPSCs by the chronic exposure to PGE2 could provide the information helpful to understand the cancer initiation connected with the chronic inflammation.

## Materials and methods

### Cell culture

Mouse induced pluripotent stem cells (miPSCs, iPS-MEF-Ng-20D-17; Lot.012) were provided by RIKEN Cell Bank (Japan). miPSCs were maintained under the humidified 5% CO_2_ atmosphere at 37 °C on feeder layer of mitomycin-C-treated mouse embryonic fibroblasts (Reprocell, Japan) in miPS medium (DMEM media (Wako, Tokyo, Japan) supplemented with 15% fetal bovine serum (FBS), 0.1 mM MEM non-essential amino acids (NEAA) (Gibco, Waltham, MA), 2 mM L-glutamine (Nacalai Tesque, Kyoto, Japan), 0.1 mM 2-mercaptoethanol (Millipore, MA), leukemia inhibitory factor (LIF, Millipore, MA), 50 U/mL penicillin/streptomycin). Then, miPSCs were transferred to 0.1% gelatin coated 60 mm-dishes in the same medium.

For primary cultured cells, the mouse allografts were excised just after euthanasia and cut into small pieces of approximately one mm^3^. After washed with Hanks' balanced salt solution (HBSS) for 3 times, the pieces were transferred into a 15-mL tube containing 4 mL of dissociation buffer consisting of 0.25% trypsin, 0.1% collagenase, 20% KnockOut Serum Replacement (Thermo Fisher Scientific, MA) and 1 mM CaCl_2_ in PBS, and the tube was incubated at 37 °C for 40 min. Then the digestion was terminated by adding 5 mL of DMEM containing 10% FBS. The suspension was centrifuged at 1000 rpm for 5 min and the cell pellet was suspended in 5 mL HBSS followed by centrifugation at 1000 rpm for 5 min. The cells were finally suspended in an appropriate volume of miPS medium without LIF and seeded at 5 × 10^5^ cells per 60-mm dish. To remove the host derived cells in the primary cultures, the cells were treated with puromycin for 24 h.

### Comprehensive phospholipids analysis

Comprehensive phospholipids analysis was described previously^40,41^. Briefly, total PLs were extracted from the exosome of LLC cells with the Bligh-Dyer method^42^. An aliquot of the lower/organic phase was evaporated to dryness under N2, and the residue was dissolved in methanol for LC/MS/MS measurements of PC and PE. To analyze PS and PI, another aliquot of the same lipid extract was added with an equal volume of methanol before being loaded onto a DEAE-cellulose column (Santa Cruz Biotechnology) pre-equilibrated with chloroform. After successive washes with chloroform/methanol (1:1, v/v), the acidic PLs were eluted with chloroform/methanol/HCl/water (12:12:1:1, v/v), followed by evaporation to dryness to give a residue, which was resolved in methanol. The resultant fraction was subjected to a methylation reaction with TMS-diazomethane before LC/MS/MS analysis^43^.

### Mass spectrometric analyses

LC-electrospray ionization-MS/MS analysis was performed with an UltiMate 3000 LC system (Thermo-Fisher Scientific, MA) equipped with HTC PAL autosampler (CTC Analytics). A 10 µL aliquot of the lipid samples was injected and the lipids were separated on Waters X-Bridge C18 column (3.5 µm, 1.0 mm × 150 mm i.d.) at room temperature (25 °C) using a gradient solvent system as follows: mobile phase A (isopropanol/methanol/water (5/1/4 v/v/v) supplemented with 5 mM ammonium formate and 0.05% ammonium hydroxide)/mobile phase B (isopropanol supplemented with 5 mM ammonium formate and 0.05% ammonium hydroxide) ratios of 70%/30% (0 min), 50%/50% (0–2 min), 20%/80% (2–13 min), 5%/95% (13–15 min), 5%/95% (15–30 min), 95%/5% (30–31 min), 95%/5% (31–35 min) and 70%/30% (35–45 min). Flow rate was 20 µL/min. PLs species was measured by the selected reaction monitoring (SRM) in positive ion mode with a triple-stage quadrupole mass spectrometer (TSQ Vantage AM, Thermo-Fisher Scientific, MA). The characteristic fragments of individual PLs were detected by the product ion scan (MS/MS mode). Chromatographic peak areas were used for comparative quantitation of each molecular species (e.g., 32:0, 34:1) in a given class of the phospholipids (e.g., PC, PE, PS, PI).

### Quantification of PGE2 and LTB4

The quantification of PGE2 was measured with the Prostaglandin E2 ELISA Kit (Cayman, MI). LTB4 quantification was quantified with the LTB4 ELISA Kit (Enzo, Switzerland). The developed colour of reaction was detected by the absorbance at 405 nm using a plate reader EnSpire2300 (PerkinElmer, MA), and PGE2 or LTB4 was quantified with a 4-parameter logistics curve of the standard.

### Conversion miPSCs in the presence PGE2

The miPSCs was feeder less and cultured in LIF-free miPS medium for 1 day, replaced with new miPS medium, and then PGE2 was added to a final concentration of 10 ng/mL. After replacement with fresh medium daily, PGE2 was added to a final concentration of 10 ng/mL. When the cells became confluent in the dish, they were passaged and seeded into a new dish. This process was repeated, and the cells were cultured for 4 weeks. Conversion of miPSCs into CSC in the presence of conditioned medium (CM) of Lewis lung carcinoma cells was basically carried out as a positive control following the method developed by our group^6,44^.

### Flow cytometric analysis

Cells were seeded in a gelatin-coated 60-mm dish. After 3 days, the cells were trypsinized and washed 3 times with PBS. The cells were analyzed on an Accuri C6 Plus flow cytometer (BD Bioscience, San Jose, CA) and then analyzed by FlowJo software excluding the patterns of cell debris and aggregates based on scatter signals.

### Protein extraction and Western blotting

The culture medium of the cells in 100-mm dish was removed and the cells were washed with PBS (–). Then sample buffer containing SDS and 2-mercaptoethanol was added, and the cells were lysed by pipetting. After brief sonication on ice, protein amount was quantified by MicroBCA (Thermo-Fischer, MA) following manufacturer’s protocol.

Each sample of 20 μg protein per lane was applied to 12.5% polyacrylamide gel, electrophoresed and blotted onto polyvinylidene fluoride (PVDF) membrane (Thermo-Fischer, MA). After blocking with 5% bovine serum albumin for 1 h, the membrane was incubated with each primary antibody overnight in a refrigerator followed by the incubation with a secondary antibody labelled with horseradish peroxidase (HRP) at room temperature for 1 h. Bound antibody was detected by substrate reagent ECL Prime (GE Healthcare, CA). The blots were cut prior to hybridization with antibodies during blotting. The protein bands in the Western blotting were subjected to densitometric quantification by ImageJ (https://imagej.nih.gov/ij/). Each quantification was made from three experiments. The whole images of the blots are listed in the supplementary Figs. S4, S5, and S6. Primary antibodies against β-Actin, p-Akt(Ser473) and Akt were from Cell Signaling Technology (MA). Those against EP-2, EP-4, cPLA2 and COX-2 were from Abcam (MA), Proteintech (IL), GeneTex (CA) and Cayman (MI), respectively. HRP labelled anti-rabbit secondary antibodies were frome Merck (Darmstadt, Germany).

### RNA extraction, real-time qPCR and mutation analysis

RNA was extracted from the collected cells in a 100-mm dish with RNAiso Plus (Takara Bio, Japan), treated with DNase I (Invitrogen, MA) and then reverse transcribed using PrimeScript RT Master Mix (Takara Bio, Japan). Afterwards, RT-qPCR was performed using Brilliant III Ultra-Fast SYBR Green QPCR Master Mix with Low ROX (Agilent Technologies, CA) and each appropriate primer set. The expression level of each gene was measured with a real-time PCR device Mx3000P (Agilent Technologies, CA). The cDNA of interest was sequenced to assess mutations with Genetic Analyzer 3130 (Applied Systems, UK) following manufacturer’s instruction. The primer sequences are listed in the Supplementary Table S2. Relative expression levels were compared with miPSCs value as one. miPSCs were cultured in miPS medium with LIF.

### RNA sequencing and bioinformatic analysis

The 150-bp paired sequencing was performed with Novaseq6000 (Illumina, CA) via (Veritas company, CA, USA). The RNA-seq data was further analyzed using the Galaxy platform (http://usegalaxy.org). The Tophat, Cuffquant, and Cuffnrorm tools were used to map, quantity, and normalize expression reads as HT-seq counts, respectively. The Cuffdiff tool was used to identify differentially expressed genes which further used as input for Kyoto Encyclopaedia of Gene and Genome (KEGG) pathway enrichment analysis at Database for Annotation^45^, Visualization and Integrated Discovery (DAVID) database http://david.ncifcrf.gov^46^. The integrated Differential Expression and Pathway analysis (iDEP) (http://bioinformatics.sdstate.edu/idep93/) was used to generate the heat map and for Parametric Gene Set Enrichment Analysis (PGSEA) (Bioconductor—PGSEA (riken.jp))^47^.

### Sphere formation and extreme limiting dilution analysis

miPS-PGE2 cells were cultured in a 60-mm low adsorption dish (Corning, NY). Serum-free DMEM containing NEAA, L-Glutamine, 2-mercaptoethanol and Insulin-transferrin-selenium-X supplemented with Pen/Strep was used for culture. For extreme limiting dilution analysis (ELDA)^48^, cells were seeded on a low-adsorption 96-well plate (IWAKI, Japan) at 1–200 cells/ well, and sphere formation was confirmed at day 5. The results were analysed with the software provided at http://bioinf.wehi.edu.au/software/elda/index.html.

### Transplantation of cells into nude mice

miPS-LLCcmP, miPS-PGE2 and miPS-PGE2P cells were pelleted by centrifugation, resuspended in 100 µl of PBS and counted with trypan blue exclusion method. Then 1X10^6^ cells were reconstituted in 100 µl of PBS and subcutaneously transplanted into 5-week-old Balb/c nu/nu female nude mice (Charles River Labs., Japan). The major axis and minor axis of tumors were measured over time, and the tumor volume was calculated by (major axis)  ×  (minor axis)^2^ × 1/2. At the end of the experiments, the mice were euthanized by the isoflurane-euthanasia method. Five percent of isoflurane (WAKO Fujifilm, Japan) was exposed to the mice and the exposure was continued until one minute after their breathing stopped. Finally, euthanasia was confirmed by cervical dislocation.

### Hematoxylin and eosin staining and immunohistochemistry

Eighteen days after transplanting the cells, developed tumors were excised, fixed in 10% formaldehyde solution (WAKO Fujifilm, Japan) for 24–48 h, and embedded in paraffin (WAKO Fujifilm, Japan). Then, the paraffin embedded tissues were sectioned by 5 μm thickness with a microtome RM2255 (LEICA, Germany) and put on slides (Plutinum Pro-11, Matsunami Glass, Japan). The tissue sections on slides were stained with 5% hematoxylin and 5% eosin (H&E) (WAKO Fujifilm, Japan). After drying, stained slides were observed and photographed under an inverted microscope CKX41 (Olympus, Japan) equipped with a digital camera.

### Tube formations assay in vitro

Matrigel (Corning, NY) was dissolved at 4 °C overnight. A sterilized cover glass (Matsunami Glass, Japan) was placed in a 12-well plate (TPP, Switzerland). Then 100 µL of Matrigel was placed on the glass and incubated at 37 °C for 30 min to gel. miPS-PGE2 cells suspended in the basic endothelial medium EGM-2 medium (Lonza Japan) were seeded on the gelled Matrigel at 2 × 10^6^ cells/well and incubated at 37 °C in 5% CO_2_ for 24 h. The formed luminal structure was observed and photographed under an inverted microscope CKX41 (Olympus, Japan) equipped with a digital camera. All of the formed tubes and networks were imaged by inverted phase-contrast microscopy. The tube branching points were counted using Image J software and statistically analyzed.

### Immunofluorescent staining

Cells were cultured on cover slip coated with Matrigel placed in 12-well plates. After 24 h tubes were photographed, washed with cold PBS and immobilized with 3.7% formaldehyde. Then the cells were washed with 0.05% Tween 20-containing phosphate buffered saline (PBS-T) and blocked with 10% FBS in PBS. Cells were incubated with the primary antibody (anti-CD31 rabbit antibody, Abcam, UK) followed by the secondary antibody (Alexa fluor 555 coupled anti-rabbit IgG goat antibody, Invitrogen, MA), fixed with soft Mont containing DAPI (Wako, Japan) and photographed under an inverted microscope equipped with fluorescent device and digital camera (CKX41 and IX70 Olympus). CD31 staining was confirmed by comparing the sample without anti-CD31 antibody treatment (Supplementary Fig. S3).

### Statistical analysis

The data were analyzed using two-tailed student’s t-test and were presented as the mean ± standard deviation (SD) at least three-time determinations. A *p*-value less than 0.1 was considered statically significant, while less than 0.01 was highly significant. In each figure where it is available, asterisks depict as * for *p* < 0.1, ** for *p* < 0.05 and *** for *p* < 0.01.

### Ethics approval and consent to participate

The plan of animal experiments in this study was reviewed and approved by the ethics committee for animal experiments of Okayama University under the IDs: OKU-2019496 (2019) and OKU-2020382 (2020). All methods in the current study were conducted in accordance with relevant guidelines and regulations and reported in accordance with ARRIVE guidelines.

## Supplementary Information

Supplementary Information.

## Abbreviations

Alox-5: Arachidonate 5-lipoxigenae
CM: Conditioned medium
COL1A1: Collagen type I α1
COL4A1: Collagen type IV α1
COL4A2: Collagen type IV α2
COX-2: Cyclooxygenase 2
cPLA2: Cytoplasmic phospholipase A2
CSC: Cancer stem cell
DAVID: Database for annotation, visualization and integrated discovery
FLNB: Filamin B
GLI2: GLI family zinc finger 2
GPCR: G-protein coupled receptor
iDEP: Integrated differential expression and pathway analysis
IGF1R: Insulin-like growth factor 1 receptor
IL-6: Interleukin 6
iPSCs: Induced pluripotent stem cells
ITGAV: Integrin subunit αV
KEGG: Kyoto encyclopedia of gene and genomes
LIF: Leukaemia inhibitory factor
LLC: Lewis lung carcinoma
LPC: Lysophosphatidylcholine
LPE: Lysophosphatidylethanolamine
LPI: Lysophosphatidylinositol
LPS: Lysophosphatidylserine
LTA4h: Leukotriene A4 hydrolase
LTB4: Leukotriene B4
MCP-1: Monocyte chemoattractant protein-1
miPSCs: Mouse induced pluripotent stem cells
miPS-LLCcm: The model of CSCs prepared from miPSCs by the treatment with the conditioned medium of LLC cells
miPS-LLCcmP: The primary cells from subcutaneously transplanted miPS-LLCcm cells
miPS-PGE2: The cells from miPSCs by the treatment with PGE2
miPS-PGE2P: The primary cells from subcutaneously transplanted miPS-PGE2 cells
PC: Phosphatidylcholine
PE: Phosphatidylethanolamine
PGES: Prostaglandin E synthase
PGSEA: Parametric gene set enrichment analysis
PGE2: Prostaglandin E2
PI: Phosphatidylinositol
PIP3: Phosphatase of phosphatidyl inositol 3,4,5 phosphate
PS: Phosphatidylserine
PTEN: Phosphatase and tensin homologue
ROS: Reactive oxygen species
Shh: Sonic hedgehog
TNF-alpha: Tumor necrosis factor alpha
VCL: Vinculin

## References

[1] Sung H (2021). Global cancer statistics 2020: GLOBOCAN Estimates of incidence and mortality worldwide for 36 cancers in 185 countries. CA Cancer J. Clin..

[2] Afify SM, Seno M (2019). Conversion of stem cells to cancer stem cells: Undercurrent of cancer initiation. Cancers (Basel)..

[3] Shiozawa Y, Nie B, Pienta KJ, Morgan TM, Taichman RS (2013). Cancer stem cells and their role in metastasis. Pharmacol. Ther..

[4] Ayob AZ, Ramasamy TS (2018). Cancer stem cells as key drivers of tumor progression. J. Biomed. Sci..

[5] Rich JN (2016). Cancer stem cells: understanding tumor hierarchy and heterogeneity. Medicine (Baltimore).

[6] Chen L (2012). A model of cancer stem cells derived from mouse induced pluripotent stem cells. PLoS ONE.

[7] Yan T (2014). Characterization of cancer stem-like cells derived from mouse induced pluripotent stem cells transformed by tumor-derived extracellular vesicles. J. Cancer..

[8] Singh N, Baby D, Rajguru JP, Patil PB, Thakkannavar SS, Pujari VB (2019). Inflammation and cancer. Ann. Afr. Med..

[9] Multhoff G, Molls M, Radons J (2012). Chroni inflammation in cancer development. Front. Immunol..

[10] Oo AKK (2018). Up-regulation of PI 3-kinases and activation of PI3K-Akt signaling pathway in cancer stem-like cells through DNA hypomethylation mediated by the cancer microenvironment. Transl. Oncol..

[11] Afify SM, Oo AKK, Hassan G, Seno A, Seno M (2021). How can we turn the PI3K/AKT/mTOR pathway down? Insights into inhibition and treatment of cancer. Expert Rev. Anticancer Ther..

[12] Hilvo M (2011). Novel theranostic opportunities offered by characterization of altered membrane lipid metabolism in breast cancer progression. Cancer Res..

[13] Samuels, Y., et al. High frequency of mutations of the PIK3CA gene in human cancers. *Science*. **304**, 554 (2004). 10.1126/science.1096502 15016963

[14] Baker RR, Thompson W (1972). Positional distribution and turnover of fatty acids in phosphatidic acid, phosphinositides, phosphatidylcholine and phosphatidylethanolamine in rat brain in vivo. Biochim. Biophys. Acta..

[15] Holub BJ, Kuksis A (1971). Structural and metabolic interrelationships among glycerophosphatides of rat liver in vivo. Can. J. Biochem..

[16] Gomes RN, Costa SF, Colquhoun A (2018). Eicosanoids and cancer. Clinics (Sao Paulo)..

[17] George RJ, Sturmoski MA, Anant S, Houchen CW (2007). EP4 mediates PGE2 dependent cell survival through the PI3 kinase/AKT pathway. Prostaglandins Other Lipid Mediat..

[18] Xu S, Zhou W, Ge J, Zhang Z (2018). Prostaglandin E2 receptor EP4 is involved in the cell growth and invasion of prostate cancer via the cAMP-PKA/PI3K-Akt signaling pathway. Mol. Med. Rep..

[19] Sun X, Li Q (2018). Prostaglandin EP2 receptor: Novel therapeutic target for human cancers (Review). Int. J. Mol. Med..

[20] Pastrana E, Silva-Vargas V, Doetsch F (2011). Eyes wide open: A critical review of sphere-formation as an assay for stem cells. Cell Stem Cell.

[21] Zhang Y (2017). NOTCH1 signaling regulates self-renewal and platinum Chemoresistance of Cancer stem-like cells in human non-small cell lung Cancer. Cancer Res..

[22] Pointer KB, Clark PA, Eliceiri KW, Salamat MS, Robertson GA, Kuo JS (2017). Administration of non-Torsadogenic human ether-a-go-go-related gene inhibitors is associated with better survival for high hERG-expressing glioblastoma patients. Clin. Cancer Res..

[23] Ioris RM (2017). SIRT6 suppresses cancer stem-like capacity in tumors with PI3K activation independently of its deacetylase activity. Cell Rep..

[24] Chang L (2015). Targeting PI3K/Akt/mTOR signaling pathway in the treatment of prostate cancer radioresistance. Crit. Rev. Oncol. Hematol..

[25] Papadimitrakopoulou V (2012). Development of PI3K/AKT/mTOR pathway inhibitors and their application in personalized therapy for non-small-cell lung cancer. J. Thorac. Oncol..

[26] Rasheed BK (1997). PTEN gene mutations are seen in high-grade but not in low-grade gliomas. Cancer Res..

[27] Risinger JI (1998). PTEN mutation in endometrial cancers is associated with favorable clinical and pathologic characteristics. Clin. Cancer Res..

[28] Rashmi R (2014). AKT inhibitors promote cell death in cervical cancer through disruption of mTOR signaling and glucose uptake. PLoS ONE.

[29] Bays LJ, DeMali AK (2017). Vinculin in cell-cell and cell-matrix adhesions. Cell Mol. Life Sci..

[30] Takafuta T, Wu G, Murphy FG, Shapiro S (1998). Human beta-filamin is a new protein that interacts with the cytoplasmic tail of glycoprotein Ibalpha. J. Biol. Chem..

[31] Bhattacharjee S (2021). Tumor restriction by type I collagen opposes tumor-promoting effects of cancer-associated fibroblasts. J. Clin. Invest..

[32] Wang D, Dubois RN (2010). Eicosanoids and cancer. Nat. Rev. Cancer..

[33] Wang D, Fu L, Sun H, Guo L, DuBois NR (2015). Prostaglandin E2 promotes colorectal cancer stem cell expansion and metastasis in mice. Gastroenterology.

[34] Navtej S, Buttar NS, Wang KK (2000). The, “Aspirin” of the new millennium: Cyclooxygenase-2 inhibitors. Mayo Clin. Proc..

[35] Garcia-Albeniz X, Chan AT (2011). Aspirin for the prevention of colorectal cancer. Best Pract. Res. Clin. Gastroenterol..

[36] Perisetti A, Goyal H, Tharian B, Inamdar S, Mehta JL (2021). Aspirin for prevention of colorectal cancer in the elderly: Friend or foe?. Ann. Gastroenterol..

[37] Sheta M (2021). Chronic exposure to FGF2 converts iPSCs into cancer stem cells with an enhanced integrin/focal adhesion/PI3K/AKT axis. Cancer Lett..

[38] Milla AL, Gonzalez-Ramirez NC, Palma V (2012). Sonic Hedgehog in cancer stem cells: A novel link with autophagy. Biol. Res..

[39] Hartog H, Van Der Graaf TW, Boezen MH, Wesseling J (2012). Treatment of breast cancer cells by IGF1R tyrosine kinase inhibitor combined with conventional systemic drugs. Anticancer Res..

[40] Imae, R., *et al.* LYCAT, a homologue of C. elegans acl-8, acl-9, and acl-10, determines the fatty acid composition of phosphatidylinositol in mice. *J. Lipid Res*. **53**, 335–347(2012). 10.1194/jlr.M018655 PMC3276457 22172515

[41] Baba T (2014). Phosphatidic acid (PA)-preferring phospholipase A1 regulates mitochondrial dynamics. J. Biol. Chem..

[42] Bligh EG, Dyer WJ (1959). A rapid method of total lipid extraction and purification. Can. J. Biochem. Physiol..

[43] Kielkowska A (2014). A new approach to measuring phosphoinositides in cells by mass spectrometry. Adv. Biol. Requl..

[44] Afify SM, Chen L, Yan T, Calle AS, Nair N, Murakami C, Zahra MH, Okada N, Iwasaki Y, Seno A, Seno M (2019). Method to convert stem cells into cancer stem cells. Methods Protoc..

[45] Kanehisa M, Goto S (2000). KEGG: Kyoto encyclopedia of genes and genomes. Nucleic Acids Res..

[46] Huang DW, Sherman BT, Lempicki RA (2009). Systematic and integrative analysis of large gene lists using DAVID Bioinformatics Resources. Nat. Protoc..

[47] Ge SX, Son EW, Yao R (2018). iDEP: An integrated web application for differential expression and pathway analysis of RNA-Seq data. BMC Bioinformatics.

[48] Hu Y, Smyth GK (2009). ELDA: Extreme limiting dilution analysis for comparing depleted and enriched populations in stem cell and other assays. J. Immunol. Methods..

