# Supplementary material for: Cancer stem cells induced by chronic stimulation with prostaglandin E2 exhibited constitutively activated PI3K axis
Source: Sci Rep. 2022 Sep 17;12:15628. doi: 10.1038/s41598-022-19265-7 (PMC9482612; doi:10.1038/s41598-022-19265-7)
Supplement: Supplementary file 1 — Supplementary Information. [file 41598_2022_19265_MOESM1_ESM.docx]

Title

**Cancer stem cells induced by chronic stimulation with prostaglandin E2 exhibited constitutively activated PI3K axis**

Authors

Hideki Minematsu^1,2^, Said M. Afify^3,4^, Yuki Sugihara^2^, Ghmkin Hassan^4^, Maram H. Zahra^4^, Akimasa Seno^4,5^, Masaki Adachi^2^, Masaharu Seno^1,4^*

Affiliations

^1^ Laboratory of Nao-Biotechnology, Division of Medical Bioengineering, Graduate School of Natural Science and Technology, Okayama University, Okayama 700-8530, Japan.

^2^ R&D Center, Katayama Chemicals Ind., Co. Ltd,. Ina, Minoh, Osaka 562-0015, Japan.

^3^Division of Biochemistry, Chemistry Department, Faculty of Science, Menoufia University, Shebin El Koum 32511, Egypt.

^4^Department of biotechnology and drug discovery, Graduate School of Interdisciplinary Science and Engineering in Health Systems, Okayama University, Okayama 700-8530, Japan.

^5^Laboratory of Natural Food & Medicine, Co., Ltd, Okayama University Incubator, Okayama, 700- 8530, Japan

* Correspondence: mseno@okayama-u.ac.jp (MS)


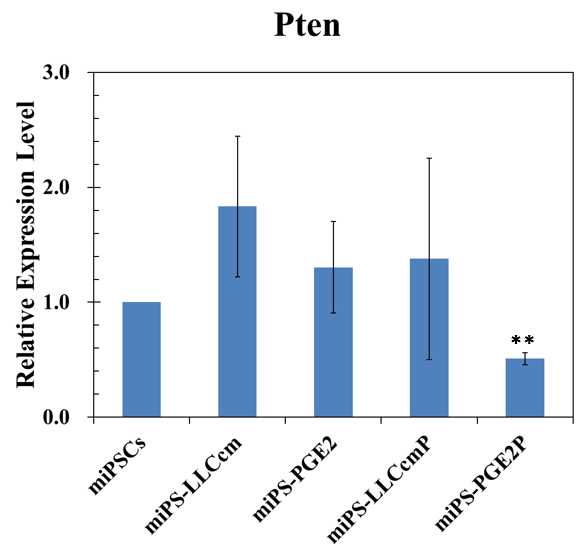


**Supplementary Figure S1.** RT-qPCR analyses of PTEN genes expression. Data are plotted as means ± SD. **P<0.05.


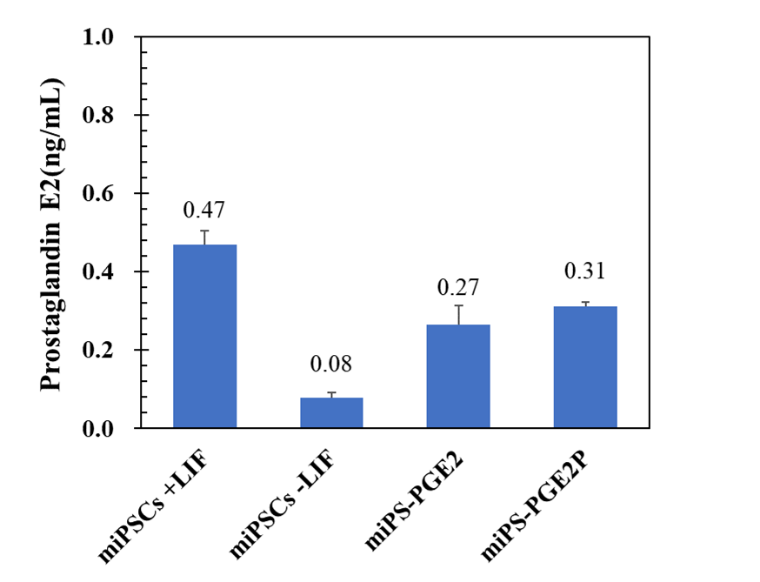


**Supplementary Figure S2.** Comparison of PGE2 production by ELISA.

**Supplementary Table S1**. List of upregulated genes and related signaling pathways in both miPS-PGE2 and miPS-PGE2P cells.

| **GENE NAME** | **mmu05200: Pathways in cancer** | **mmu0451: Focal adhesion** | **mmu0451: ECM-receptor interaction** | **mmu0415: PI3K-Akt signaling pathway** | **mmu0439: Hippo signaling pathway** | **mmu0431: Wnt signaling pathway** | **HTseq-counts** | | |
| --- | --- | --- | --- | --- | --- | --- | --- | --- | --- |
|  |  |  |  |  |  |  | **miPS** | **miPS-PGE2** | **miPS-PGE2P** |
| AXIN2 |  |  |  |  | **X*^1^** | **X** | 3.0 | 46.6 | 84.6 |
| BMPR2 |  |  |  | **X** |  |  | 17.0 | 130.0 | 107.7 |
| CBL | **X** |  |  |  |  |  | 4.0 | 112.1 | 68.8 |
| CCND2 |  | **X** |  | **X** |  | **X** | 4.0 | 78.5 | 113.8 |
| CD44 |  |  | **X** |  |  |  | 0.0 | 26.5 | 51.8 |
| CTGF |  |  |  | **X** |  |  | 22.0 | 134.3 | 144.4 |
| CDKN2A | **X** |  |  |  |  |  | 3.0 | 77.4 | 52.9 |
| **COL1A1*^2^** |  | **X** | **X** |  |  |  | **0.0** | **192.8** | **395.7** |
| COL3A1 |  | **X** | **X** |  |  |  | 0.0 | 92.6 | 238.7 |
| **COL4A1** | **X** | **X** | **X** |  |  |  | **17.0** | **553.0** | **537.6** |
| **COL4A2** | **X** | **X** | **X** | **X** |  |  | **3.0** | **336.3** | **328.0** |
| COL5A1 |  | **X** | **X** | **X** |  |  | 8.0 | 177.6 | 363.3 |
| EFNB2 |  |  |  |  |  | **X** | 9.0 | 135.4 | 115.2 |
| EPHA1 |  |  |  |  |  | **X** | 7.0 | 37.4 | 91.8 |
| FGF5 |  |  |  | **X** |  |  | 0.0 | 23.8 | 59.8 |
| **FLNB** |  | **X** |  |  |  |  | **154.0** | **1675.1** | **1428.0** |
| FLNC |  | **X** |  |  |  |  | 20.0 | 197.1 | 211.7 |
| FZD2 |  |  |  |  | **X** |  | 4.0 | 62.3 | 63.0 |
| **GLI2** | **X** |  |  |  | **X** |  | **114.0** | **1498.6** | **890.0** |
| **IGF1R** | **X** | **X** |  | **X** |  |  | **22.0** | **285.4** | **177.1** |
| INSR |  |  |  | **X** |  |  | 5.0 | 104.5 | 65.9 |
| **ITGAV** | **X** | **X** | **X** | **X** |  |  | **16.0** | **270.3** | **164.5** |
| JUN |  | **X** |  |  |  | **X** | 6.0 | 63.4 | 58.3 |
| LATS2 |  |  |  |  | **X** |  | 1.0 | 93.2 | 52.6 |
| MMP2 | **X** |  |  |  |  |  | 7.0 | 144.6 | 119.2 |
| PARVA |  | **X** |  |  |  |  | 4.0 | 74.2 | 70.9 |
| PRICKLE1 |  |  |  |  |  | **X** | 1.0 | 35.2 | 63.0 |
| PTCH1 | **X** |  |  |  |  |  | 8.0 | 410.0 | 183.3 |
| SERPINE1 |  |  |  |  | **X** |  | 1.0 | 47.1 | 72.0 |
| SFRP2 |  |  |  |  |  | **X** | 2.0 | 46.6 | 31.0 |
| TGFB2 | **X** |  |  |  | **X** |  | 0.0 | 42.2 | 65.9 |
| THBS1 |  | **X** | **X** | **X** |  |  | 0.0 | 130.0 | 303.2 |
| TNC |  |  | **X** | **X** |  |  | 0.0 | 36.8 | 250.2 |
| **VCL** |  | **X** |  |  |  |  | **48.0** | **533.5** | **525.3** |
| WNT4 | **X** |  |  |  | **X** | **X** | 1.0 | 53.1 | 50.0 |
| WNT5A |  |  |  |  | **X** |  | 0.0 | 26.0 | 51.8 |
| ZYX |  | **X** |  |  |  |  | 23.0 | 114.3 | 191.2 |
| **GAPDH*^3^** |  |  |  |  |  |  | **288.0** | **261.0** | **240.2** |

*^1^: The genes nominated as positive in each pathway by KEGG analysis.

*^2^: Each gene upregulated in both miPS-PGE2 and miPS-PGE2P cells is depicted in bold together with its HTseq-counts.

*^3^: GAPDH is put as a reference of the equivalency.

**Supplementary Table S2**. List of Primers Used in the Experiments

| No. | Names | Forword Primer Sequence | Forword Primer Sequence |
| --- | --- | --- | --- |
| 1 | PLA2 | TGGCTCACAGAATAAAGGCTCTACA | GCTCACCAAGGCCATTAGCA |
| 2 | COX-2 | CTGGAACATGGACTCACTCAGTTTG | AGGCCTTTGCCACTGCTTGTA |
| 3 | PGES1 | TACGCGGTGGCTGTCATCA | CTCCACATCTGGGTCACTCCTG |
| 4 | PGES2 | CAGCCAACAAGTGGGTGACAG | GGATGTGTGAGTGTCGCATCAG |
| 5 | PGES3 | GGTGATGAGGATGTAGATTTACCAG | TGACAACAGCCCTTACTCCAGA |
| 6 | Alox-5 | GCAGATCGTGGATACTCTACCAGAC | CCTCTGGGTACATGCCTAGAAACA |
| 7 | Lta4h | GGCCCTAAAGATGGCAACTGAA | GATTTGTCGAAGGCAGCGAGA |
| 8 | Nanog | AGGGTCTGCTACTGAGATGCTCTG | AACCCAAGCACGTATCAGGG |
| 9 | Oct3/4 | TCTTTCCACCAGGCCCCCGGCTC | TGCGGGCGGACATGGGGAGATCC |
| 10 | Sox2 | TAGAGCTAGACTCCGGGCGATGA | TTGCCTTAAACAAGACCACGAAA |
| 11 | Klf4 | GGACTTACAAAATGCCAAGGGGTG | TCGCTTCCTCTTCCTCCGACACA |
| 12 | CD44 | AGAAAAATGGCCGCTACAGTATC | TGCATGTTTCAAAACCCTTGC |
| 13 | CD133 | CCTTGTGGTTCTTACGTTTGTTG | CGTTGACGACATTCTCAAGCTG |
| 14 | Pik3ca | GCCACAGACACTACTGCGT | CACCGAACAGCAAAACTCCG |
| 15 | Pik3cb | CTGATTTTACGGCGGCATGG | TGAGGGCCTCGTCAAACTTC |
| 16 | Pik3cg | ACCTGTGCCTTCTGCCTTAC | TGCGGCCTGAAACTTTTCTTC |
| 17 | Pik3r1 | AGCGGAGAACCTATTGCGAG | ACTTCGCGTCTACCACTAC |
| 18 | Pik3r5 | AAGTCCTTTGTCAGCAGTCCC | CTGGTAAACCTGCAGCAACAC |
| 19 | Pik3ap1 | GAAGGCCATTTCTGAAGATTCTGG | TCTCGTCCAGCTTGCATCTC |
| 20 | Pten | TGAAGACCATAACCCACCACAG | AGCATCTTGTTCTGTTTGTGGAAG |
| 21 | GAPDH | AACGGCACAGTCAAGGCCGA | ACCCTTTTGGCTCCACCCTT |
| 22 | Pik3ca (Sequencing1) | ACCCTATTGGTGTTACTGGGTC | TCCATGGCTTGCTCTGGTTT |
| 23 | Pik3ca (Sequencing2) | CTGCGTGGCAACCTTTATCTT | CCCAGCTCCCATCTCAGTTCA |
| 24 | Pten (Sequencing) | TGAAGACCATAACCCACCACAG | AGCATCTTGTTCTGTTTGTGGAAG |


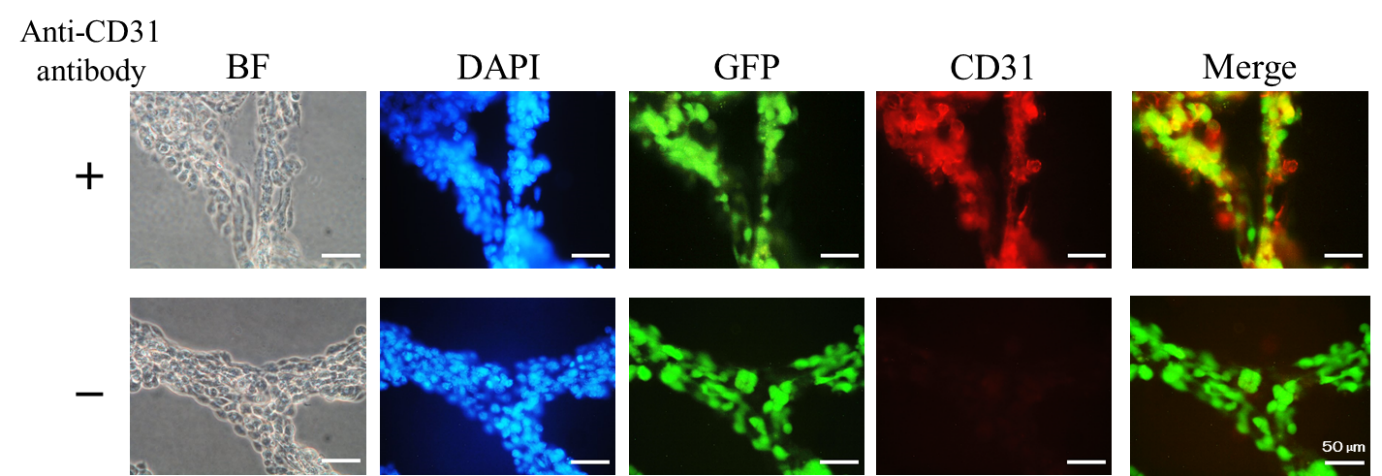


**Supplementary Figure S3.** Immunofluorescent analyses of tubes formed from miPS-PGE2P. BF, bright field. DAPI, staining for nucleus. GFP, fluorescence of GFP. CD31, staining with anti-CD31 antibody labelled with Alexa fluor 555 or without anti-CD31 antibody. Scale bars = 50.


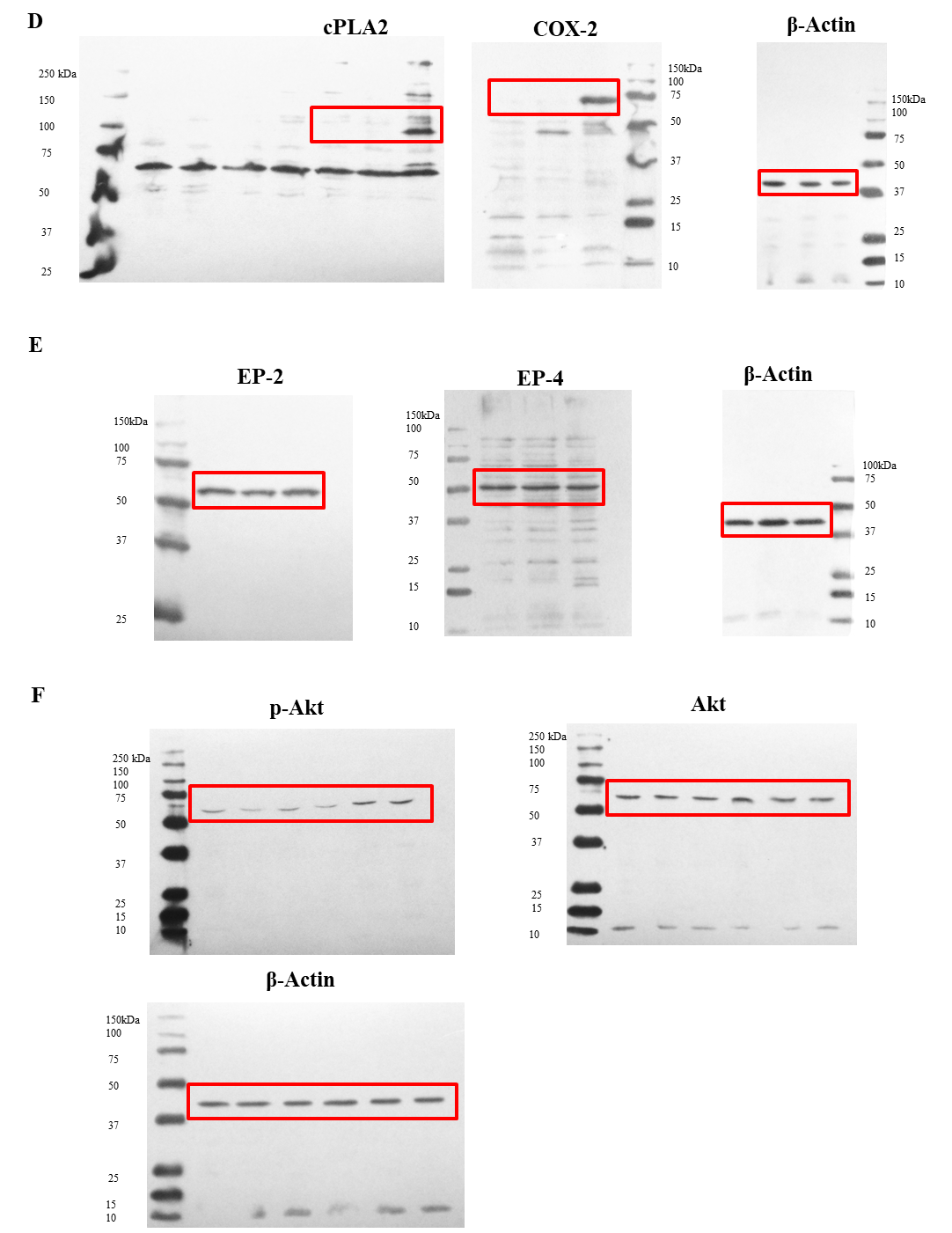


**Supplementary Figure S4.** Whole blotting images in Figure 1. The blots were cut prior to detection with antibodies during blotting.


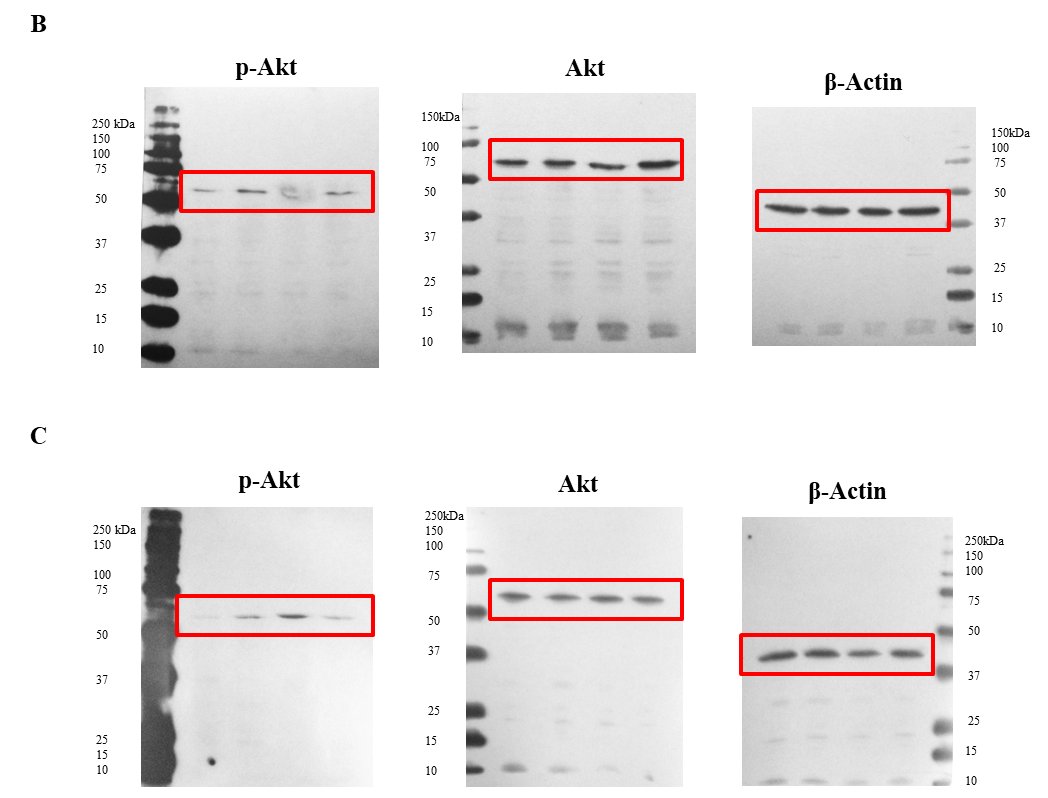


**Supplementary Figure S5.** Whole blotting images in Figure 5. The blots were cut prior to detection with antibodies during blotting.


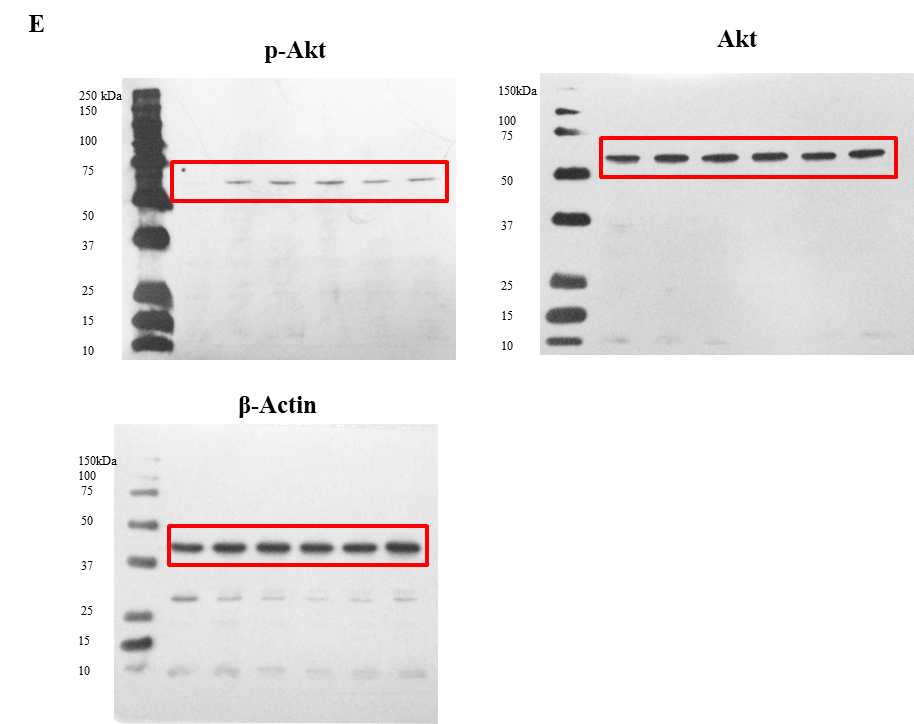


**Supplementary Figure S6.** Whole blotting images in Figure 7E. The blots were cut prior to detection with antibodies during blotting.

**
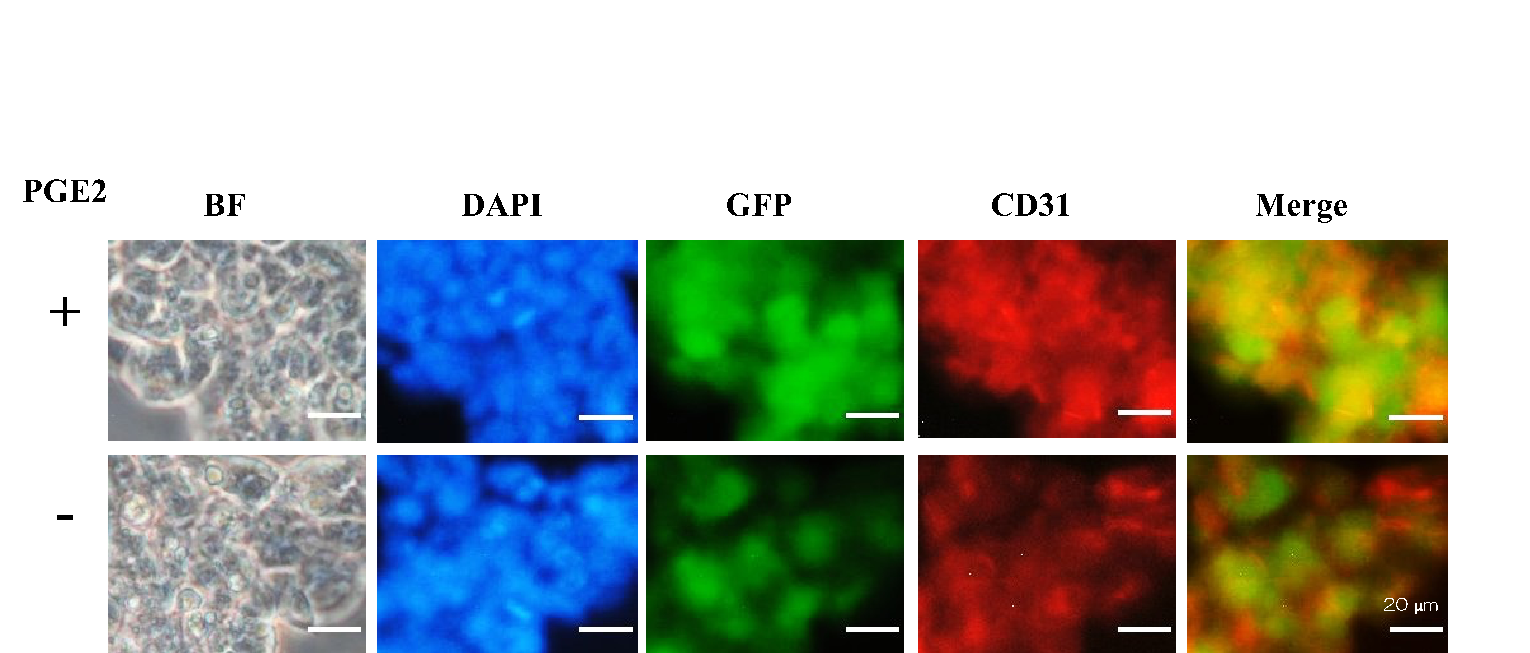
**

**Supplementary Figure S7.** Immunofluorescent analyses of tubes in BF, bright field. DAPI, staining for nucleus. GFP, fluorescence of GFP. CD31, staining with anti-CD31 antibody labelled with Alexa fluor 555. Scale bars = 20 µm.
